# Co-expression network analysis of lncRNAs and mRNAs in rat liver tissue reveals the complex interactions in response to pathogenic cytotoxicity

**DOI:** 10.7150/ijbs.33735

**Published:** 2019-08-22

**Authors:** Dongliang Leng, Chen Huang, Kuan Cheok Lei, Shixue Sun, Baoqing Sun, Xiaohua Douglas Zhang

**Affiliations:** 1 Faculty of Health Sciences, University of Macau, Taipa, Macau; 2 State Key Laboratory of Respiratory Disease, First Affiliated Hospital of Guangzhou Medical University, Guangzhou, China

**Keywords:** LncRNAs, interaction network, cytotoxicity, liver disease

## Abstract

Liver is one of the most vital organs to maintain homeostasis because of its peculiar detoxification functionalities to detoxify chemicals and metabolize drugs and toxins. Due to its crucial functions, the liver is also prone to various diseases, i.e., hepatitis, cirrhosis and hepatoma, etc. Additionally, long non-coding RNAs (lncRNAs) has emerged as key regulators which are found to play important roles in transcription, splicing, translation, replication, chromatin shaping and post translational modification of proteins in living cells. However, the underlying mechanisms of biological processes mediated by lncRNA remain unclear. Here, with the aim of disclosing potential lncRNAs implicated in the biological processes in liver in response to cytotoxicity, we performed a co-expression network analysis based on the transcriptome data of the damaged liver tissue of *Rattus norvegicus* induced by three cytotoxic compounds (carbon tetrachloride, chloroform and thioacetamide). Our analysis unveils that many biological processes and pathways were collectively affected by the three cytotoxic compounds, including drug metabolism, oxidation-reduction process, oxidative stress, glucuronidation, liver development and flavonoid biosynthetic process, etc. Also, our network analysis has identified several highly conserved lncRNA-mRNA interactions participating in those correlated processes and pathways, implying their potential roles in response to the induced cytotoxicity in liver. Our study provides new insights into lncRNA-mRNA regulatory mechanisms in response to pathogenic cytotoxic damaging in liver and facilitates the development of lncRNA-oriented therapies for hepatic diseases in the future.

## Introduction

The liver is the central organ for various physiological processes, including macronutrient metabolism, immune system support, lipid and cholesterol homeostasis, as well as the breakdown of xenobiotic compounds, i.e. drugs. However, these properties render the liver a vulnerable for pathogenesis. Despites the majority of pathogens can be eliminated or controlled by the innate and adaptive immune responses, there are still a few specific pathogens which can overpower the immune system and cause substantial morbidity and mortality worldwide. According to the statistics of Centers for Disease Control and Prevention of United States, the death rates for chronic liver disease and cirrhosis increased significantly for persons aged ≥ 45 years from 2000 to 2015^1^. Therefore, the harm of liver diseases to human beings should not be underestimated.

Hepatocytes are fragile to the damage induced by cytotoxicity. Liver intoxicated with toxins can easily result in a series of diseases, initially hepatitis, then developing into cirrhosis, finally deteriorating into hepatocellular carcinoma. The source of cytotoxin production is the reactive oxygen species occurring in the aerobic respiration. ROS have been studied for a long time, such as oxygen ions, peroxides and oxygen free radicals. Medium and high concentrations of ROS induce apoptosis through cell oxidative stress and sometimes even lead to necrosis. Recent studies have shown that ROS can bi-directionally regulate apoptosis and proliferation of some cancer cells, and the intrinsic relationship between free radicals and cell signal transduction has been found^2^. Usually, cells reduce ROS damage by enzymes such as superoxide dismutase. Some small molecules, such as vitamin C, vitamin E, uric acid and glutathione, also act as cell antioxidants^2^.

ROS can cause irreversible damage to tissues. In biological experiments, halobenzene compounds are often used as inducer to model the toxicity of cytotoxins such as carbon trichloride, carbon tetrachloride and thioacetamide. Studies have shown that the mode of toxic action of carbon trichloride and carbon tetrachloride is the formation of ROS during the metabolism of these two chlorides, resulting in damages to the body^3^, ^4^. As an important organ for the metabolism of exogenous toxic substances, liver is very sensitive to toxic damages, so toxin-induced liver disease model has become an important means of scientific research. For example, Thioacetamide-induced liver disease model was used and proteomic analysis revealed that 59 important proteins have different changes during the pathogenesis^5^.

On the other hand, lncRNA is defined as the RNA of length greater than 200 nucleotides and lacking capability to encode proteins^6^. LncRNA originates from the vast regions of non-coding DNA sequence which was once considered junk DNA. However, increasing evidence indicates that lncRNAs as a curial regulatory layer play important roles in a variety of biological processes, including transcription, splicing, translation, replication, chromatin shaping and post-translational modification, etc. 7-9. Concretely, a lncRNA named lncLSTR is found to be specifically expressed in liver and is demonstrated as a triglyceride regulator to regulate the energy metabolism in liver 10. HULC is found to be overexpressed in liver tumor and has been considered as an effective biomarker in many human cancers 11. Furthermore, a novel lncRNA named Lnc-HC is demonstrated to bind to hnRNPA2B1 in hepatocytes and can negatively regulate cholesterol metabolism^12^. Notably, most of the studies on lncRNA in liver focus on its metabolic functions, a few concerns their potential roles in liver detoxification. In the present study, aiming at discovering novel lncRNAs as well as their potential roles as antidote in liver, we performed a comprehensive transcriptomic analysis on the deep RNA sequencing data of the damaged *Rattus norvegicus* liver tissue induced by three different cytotoxic compounds (carbon tetrachloride, chloroform, thioacetamide).

## Results

### Transcriptome re-construction

In this study, three different cytotoxic chemicals were employed for treatment groups, while control groups were prepared separately. All samples were sequenced by Illumina Hiseq 2000 platform, generating a total of 1,302,411,914 reads. In order to obtain a high-confidence dataset, we filtered out low-quality reads, over-represented sequences, and adapter sequences from our raw data using Trimmomatic. 891,896,778 clean reads were yielded in carbon tetrachloride treatment, 922,721,370 in chloroform treatment and 194,462,724 in thioacetamide treatment (Table S1).

After quality control, the clean reads were subsequently aligned to a reference genome. The software STAR with 2-pass mode was chosen to align filtered reads against *R. norgegicus* reference (Ensembl release 92). As shown in Table S2, most clean reads (86% ~ 90%) could be mapped to the reference.

After alignment, reads from different libraries were pooled together for each chemical group. And mapped reads were assembled by StringTie to generate the consensus transcriptome for each chemical group. Concretely, 52001 transcripts were identified in CAR treatment group, 52,254 in CHL treatment group and 52,744 in THI treatment group. After merging the three groups, 64938 were obtained, totally (Table S3). Lastly, a visualization tool for transcriptional landscape of alternative splicing (ASTALAVISTA) was selected to search for five main alternative splicing modes, including intron retention, exon skipping, alternative 3'-acceptor, alternative 5'-donor and mutually exclusive exon. The result illustrated almost uniform splicing modes across all the groups treated by three different chemicals (Fig. S1).

### Identification of lncRNAs

**Figure 1:**
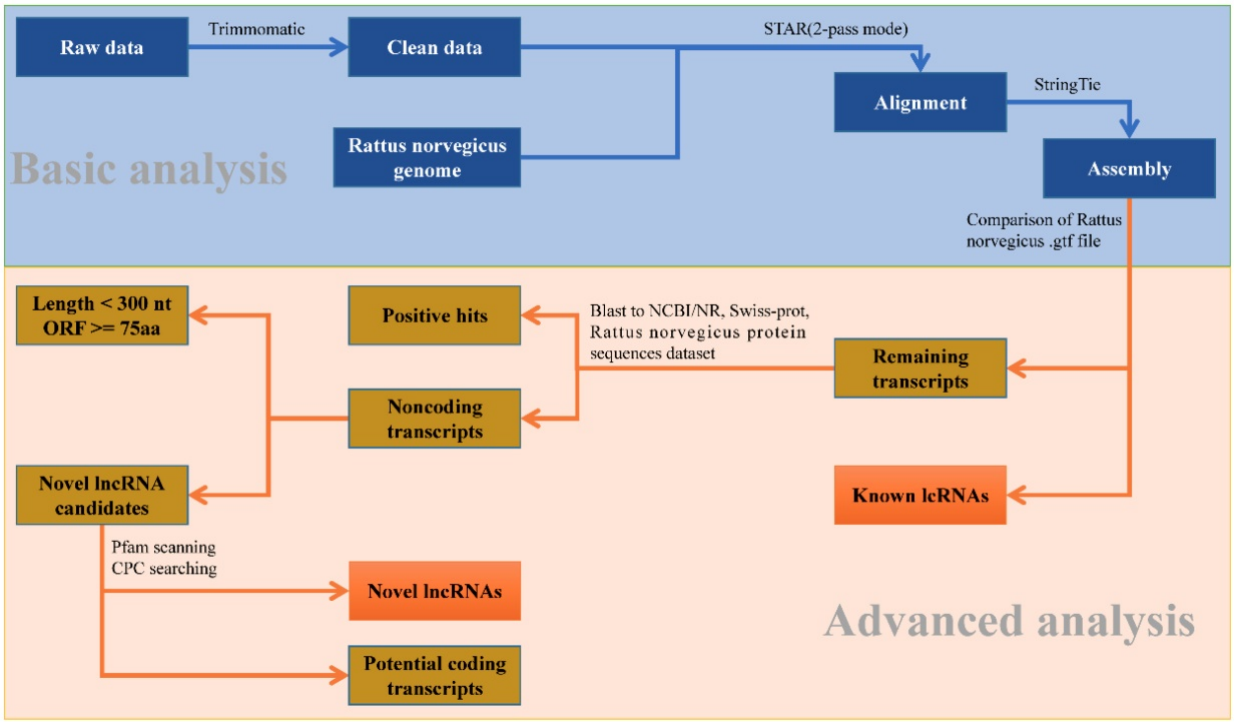
The analysis pipeline used for the identification of lncRNAs in the transcriptome of *Rattus norvegicus*.

To identify the putative lncRNAs present in each chemical treatment group, a stringent stepwise pipeline was developed to discard transcripts with evidence of protein coding potential (Fig. 1). After merging all the assembled transcripts into 64,938 transcripts through StringTie, we used in-house perl script to compare them with the lncRNAs information from gene annotation of *R. norvegicus*. 4,581 known lncRNAs with lengths larger than 200 nt and types 'antisense', 'non-coding', 'processed transcript' and 'lincRNA' were extracted. Similarly, 29,095 known mRNAs also could be identified.

Then the potential coding transcripts were queried using BLASTx algorithm against Uniport database, NCBI non-redundant protein database and protein sequences database of *R. norvegicus* derived from Ensembl database for the remaining transcripts. The result showed that 20,392 transcripts could be well compared. Furthermore, 6,820 transcripts were retained after discarding the transcripts with length less than 200 nucleotides and longest ORF greater than 100 amino acids. To effectively distinguish protein-coding and non-coding sequences, coding potential filtering was performed subsequently according to CPC and Pfam Scan (v1.3) (Table S4). Finally, 6,818 novel lncRNAs were obtained (Fig. S2-B). Combined with 4,581 known lncRNAs (Fig. S2-A) obtained from the previous analysis, a total of 11,397 lncRNAs were identified for further study (Fig. S3).

### Characteristics of lncRNAs among three differential chemical treatment groups

**Figure 2:**
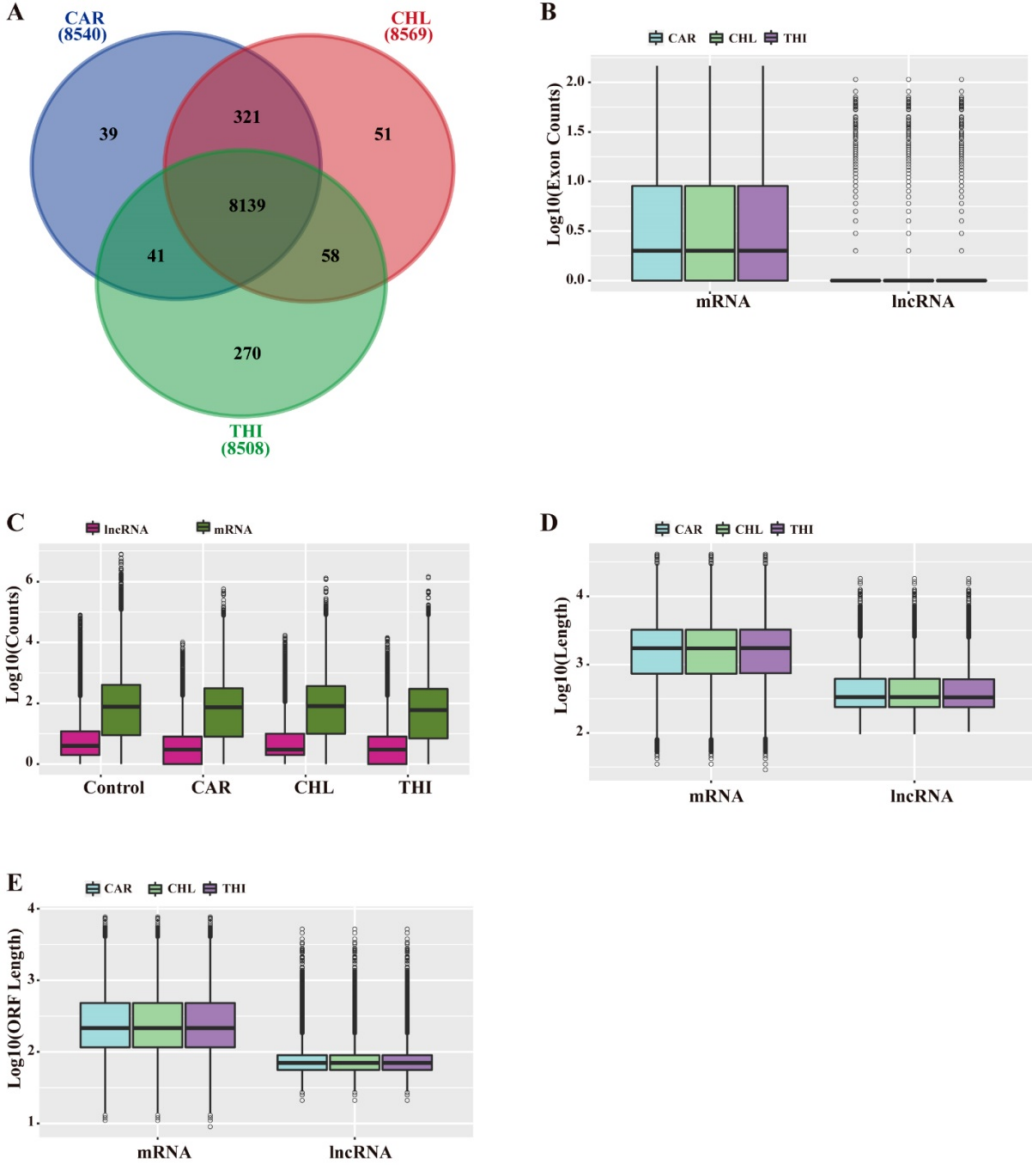
** General characteristics of lncRNAs compared to mRNAs in *Rattus norvegicus*. (A)** Relationship of lncRNAs among three chemical treatment groups. **(B)** Distribution of exon counts by log10 **(C)** Expression level indicated by log10 (normalization read counts+1). **(D)** Distribution of transcript length by log10. (E) Distribution of ORF length by log10.

Based on the above analysis, a total of 8,540, 8,569, 8,508 lncRNAs were identified in the transcriptome of CAR, CHL and THI treatment group respectively (Fig. 2-A). The features of lncRNAs were analyzed according to the following aspects, including length and their expression levels, comparing with those of mRNAs for each chemical treatment group. The results indicated that the length of lncRNAs and ORF as well as the number of exons in the three chemical treatment groups are all smaller than those of their respective mRNAs (Fig. 2-B, D, E). Furthermore, the expression of lncRNAs is also lower than that of mRNAs (Fig. 2-C).

### Differential expression of mRNAs and lncRNAs response to three different cytotoxicity-inducing chemicals

**Figure 3:**
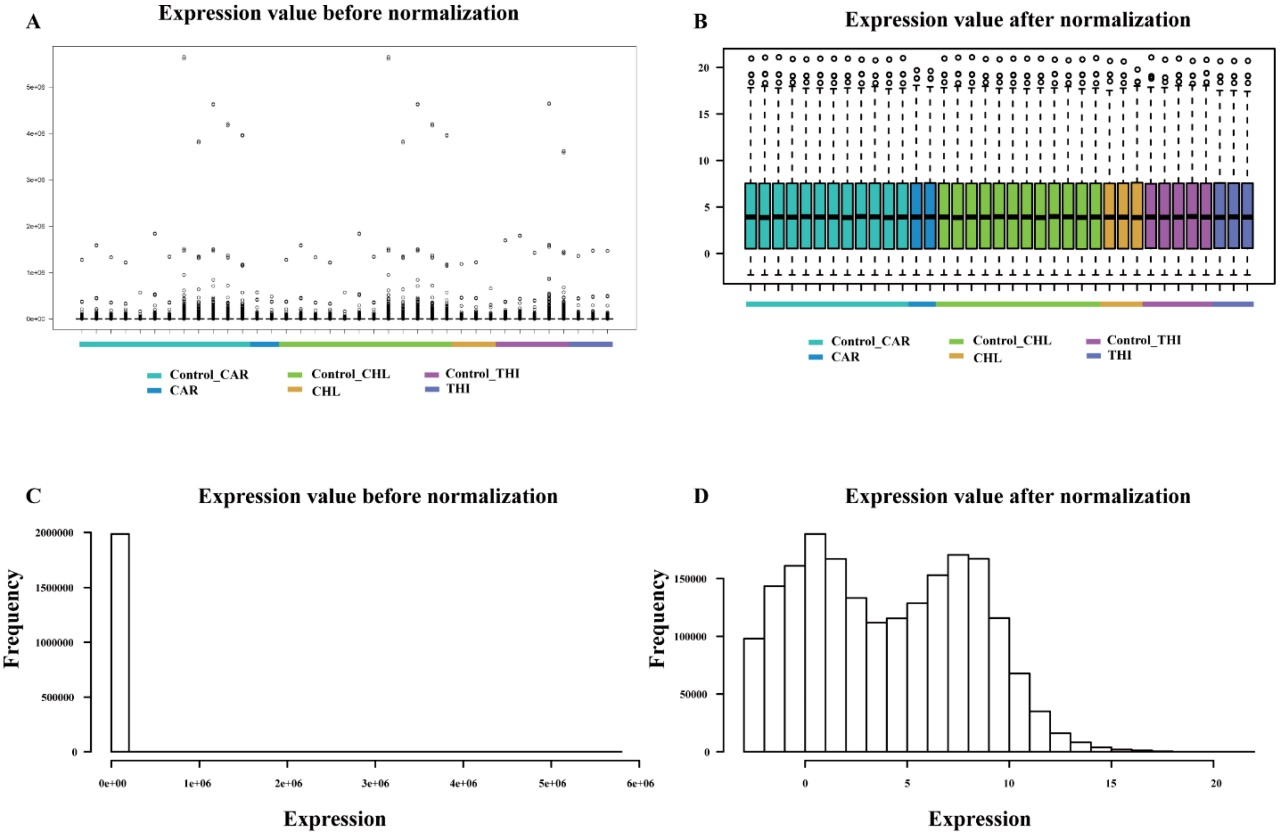
** Comparison of expression value between before normalization and after normalization. (A)** distribution of expression value before normalization. **(B)** distribution of expression value after normalization. **(C)** frequency of expression value before normalization. **(D)** frequency of expression value after normalization.

**Figure 4:**
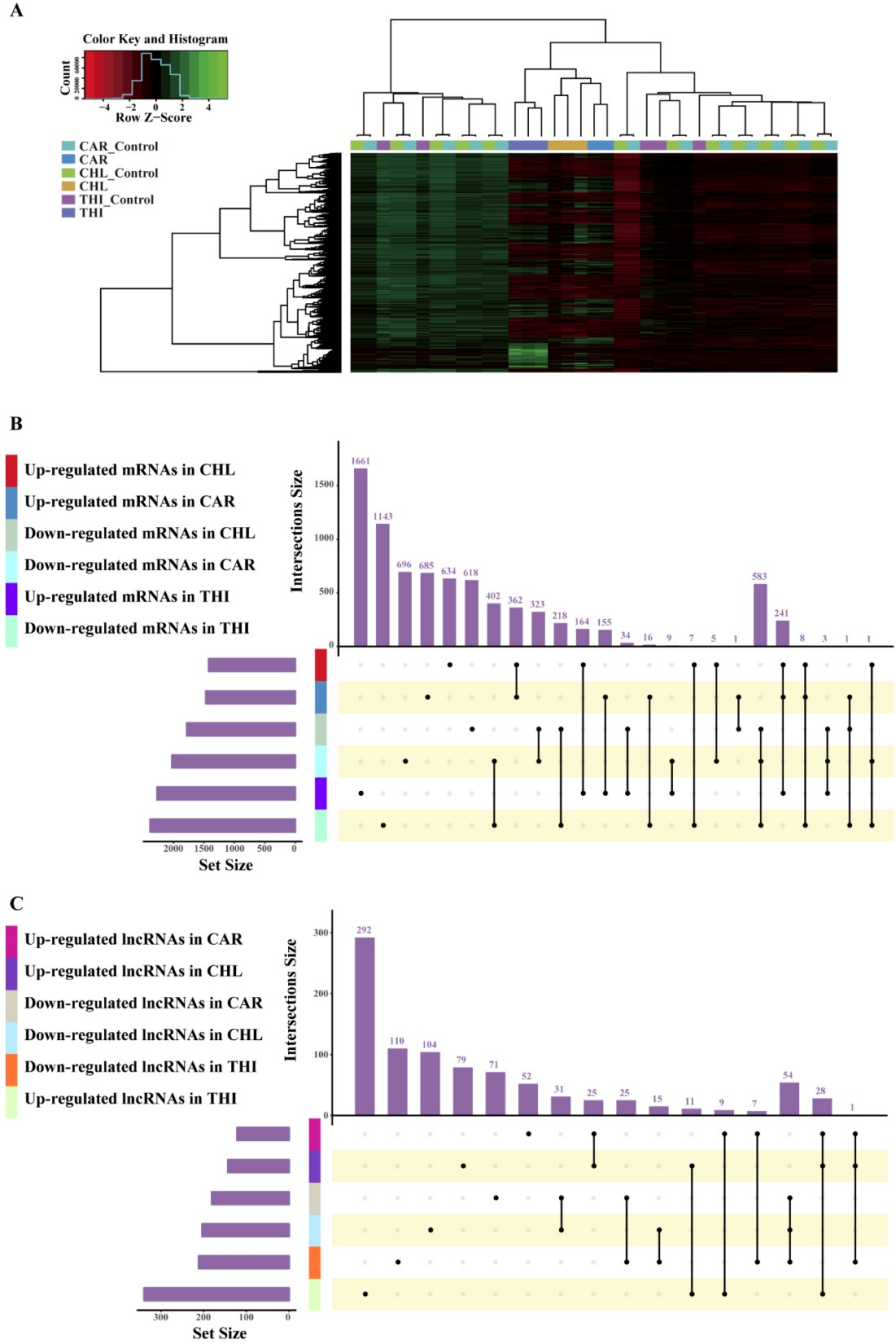
** Differentially expressed analysis in three chemical treatment groups. (A)** Heatmap of differential transcriptome expression profiles cross all the samples used in three chemical treatment. **(B)** Overlap of differential expression mRNAs among different chemical treatment. **(C)** Overlap of differential expression lncRNAs among different chemical treatment.

The goal of differential expression analysis is to investigate the lncRNAs and mRNAs that have changed significantly in abundance between case and control. Initially, the expressions of lncRNAs and mRNAs were quantified in the previous analysis. Then, we normalized the read counts for each sample (Fig. 3) and conducted the differential expression analysis using R package DESeq2 (Fig. 4-A). The detail interrelationship of differentially expressed lncRNAs and mRNAs between three chemical treatment groups was visualized in Fig. 4. Concretely, there were 303 significantly differentially expressed lncRNAs and 3,491 SDE mRNAs identified (p < 0.05 & fold change > 2) in CAR treatment group, 344 SDE lncRNAs and 3,203 SDE mRNAs identified (p < 0.05 & fold change > 2) in CHL treatment group, 552 SDE lncRNAs and 4,646 SDE mRNAs identified (p < 0.05 & fold change > 2) in THI treatment group, in comparison with their respective control group. Among these, 28 lncRNAs and 241 mRNAs were commonly upregulated, 54 lncRNAs and 583 mRNAs were commonly downregulated in three different chemical treatment groups (Fig. 4-B, C). All the differentially expressed transcripts for three comparisons was summarized in Table S5.

### WGCNA analysis reveals complex network implicated in cytotoxicity

**Figure 5:**
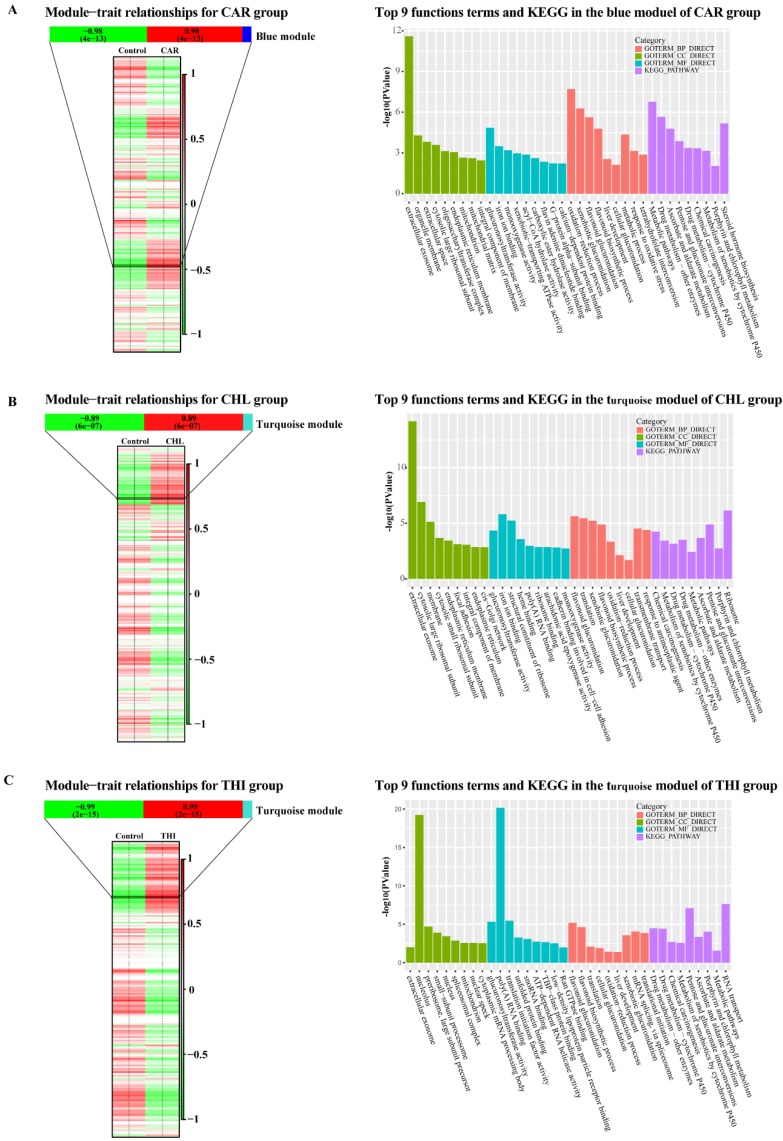
** Module-trait relationships in three chemical treatment groups and top 9 functional enrichment cross three categories together with KEGG analysis through the most related module in three chemical groups. (A)** Module-trait relationships and top 9 functional enrichment cross three categories together with KEGG analysis through the blue module in CAR group. **(B)** Module-trait relationships and top 9 functional enrichment cross three categories together with KEGG analysis through the blue module in CHL group. **(C)** Module-trait relationships and top 9 functional enrichment cross three categories together with KEGG analysis through the blue module in THI group.

WGCNA is used to extract gene modules related to traits or clinical features, and to analyze biological processes such as basic metabolic pathways, transcriptional regulation pathways and translational regulation. In this part, the WGCNA analysis was performed for each group. Firstly, normalized expression matrices were generated using DESeq2. The difference between treatment and control in each group could be clearly shown (Fig. S4). Then, the outlier samples of each group were detected from the sample clustering result (Fig. S5). There were only two outliers in group CHL in the initial clustering samples (Fig. S5-B). After removing the outliers, the appropriate soft threshold values in each group were estimated (Fig. S6). The soft threshold values which support the scale-free network atlas structure R^2^ reaching 0.8 and the average connectivity higher than 100 should be the best parameter for further analysis. In addition, the values should be an integer smaller than 15 in undirected network or smaller than 30 in directed network. After applying the function *pickSoftThreshold* in WGCNA package, 4,5 and 3 were estimated as the best soft threshold values in CAR, CHL and THI groups respectively. Substantially, giving the best soft threshold values to the parameter of power, the One-step network construction and module detection were performed using the function *blockwiseModules*. In this step, the expressed transcripts in each group were clustered into some modules according to their normalized reads count. The results showed that 265 modules, 292 modules and 178 modules were identified in CAR group, CHL group and THI group, respectively (Fig. S7). Every module shows consistent expression pattern, but the module which is strongly related to the properties should be given more attention; the reason can be strongly supported from the results of the correlation between module membership and gene significance (Fig. S8), and the clustering illustrates the relationship between module and trait among three groups (Fig. S9 - S11). The matrices of properties were designed for each group and the correlation between modules and properties were calculated (Fig. 5). In the CAR group, the blue module with 2412 transcripts has the strongest correlation with PCC = 0.98 and its p-value = 4e^-13^ (Fig. 5-A). Similarly, the turquoise module with 3965 transcripts is detected with PCC = 0.89 and p-value = 6e^-7^ in CHL group, and the turquoise module with 7923 transcripts is detected with PCC = 0.99 and p-value = 2e^-15^ in THI group (Fig 5-B, C). In order to explore the functions related to these modules, we performed Gene Ontology analysis for the modules which are most strongly related to the properties in each group, including the functions on biological process, cellular component, molecular function and KEGG pathway. There are some significant pathways enriched in each group. Concretely, there are 66 terms about the biological process, 19 terms about cellular component, 27 terms about molecular function and 26 pathways enriched in CAR group (Fig. S12 - S15). Similarly, 61 terms about biological, 26 terms about cellular component, 34 terms about molecular function and 17 pathways enriched in CHL group (Fig. S16 - S19), 54 terms about biological process, 41 terms about cellular component, 29 terms about molecular function and 15 pathways enriched in THI group (Fig. S20 - S23). This result completely showed the complex effect of cytotoxicity in rat. Afterwards, the commonly enriched functions and pathways in three groups and the common genes in these enriched functions were extracted for further analysis (Table 1).

**Figure 6:**
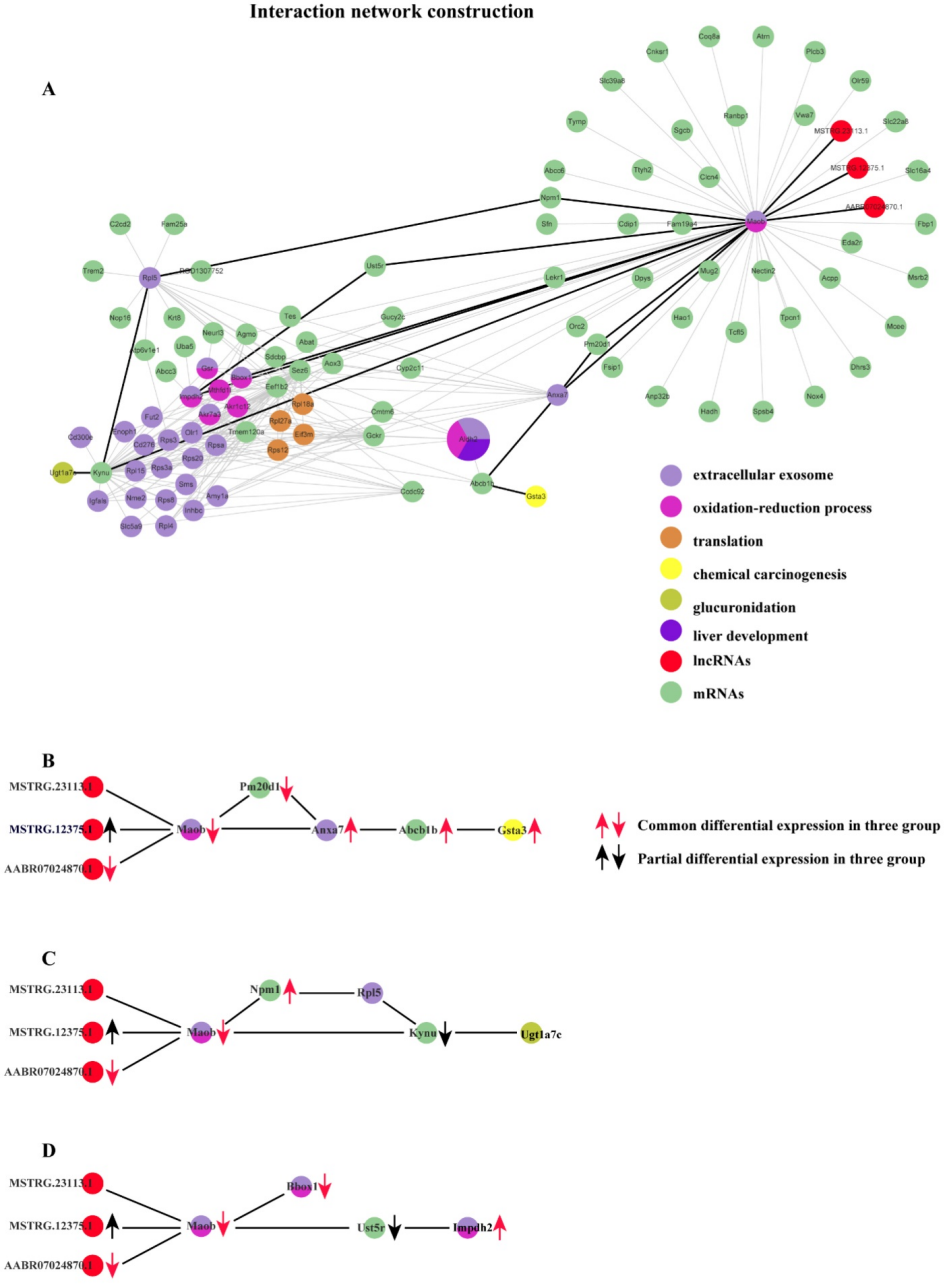
** Co-expression network constructed with the common interactions with top 20% weighted value among three groups and the important interactions related to 3 basic functions in the process of cytotoxicity. (A)** Co-expression network constructed with the common interactions with top 20% weighted value among three groups. **(B)** The important interactions related to chemical carcinogenesis. (c) The important interactions related to glucuronidation. (d) The important interactions related to extracellular exosome and oxidation-reduction process.

Based on the co-expression matrix and the best soft threshold value in each group, the co-expression networks were constructed, and TOM similarity was generated using WGCNA. The TOM similarity reflects the potential interactions between transcripts. An assumption that some pairs of interactions containing the common transcripts transcribed from the common genes identified should be given more attentions. Thus, the interactions which are consistently found to be in common in the modules which are most strongly related to the properties in each group would be filtered out. Therefore, 86320 pairs of common interactions in all three groups were collected. To focus on the key genes identified issn the common functions enriched in three groups, mRNA-mRNA and mRNA-lncRNA interaction pairs would be retained. After filtering, 16195 pairs of common interactions were located. Undeniably, there exist some false positives in the filtered common interactions. So, a higher standard should be followed. The top 20% of weighted values of the common interactions in three group simultaneously were retained to unearth the main important interactions induced by cytotoxicity. Finally, 282 common interactions with strict filtering criteria were obtained, including 3 lncRNAs, 34 mRNAs with already known functions enriched and 65 mRNAs with un-belonging to anyone of the common functions enriched (Fig. 6).

## Discussion

We performed differential expression analysis and the result shows that distinct lncRNAs and mRNAs are differentially expressed under the induction of the three compounds. Furthermore, in order to explore possible roles of those specific lncRNAs implicated in liver cytotoxicity, we utilized a co-expression network analysis to analyze the expression profile of all lncRNAs and mRNAs for each group based on WGCNA package in R. For each group, the modules which are closely related to the compound treatment were figured out. Subsequently, functional enrichment analysis based on the RNAs derived from each module were conducted, the result of which suggested diverse biological processes and pathways are affected in rat liver by different compounds. For example, small GTPase-mediated signal transduction, Golgi vesicle transport and response to thyroxine are found to be specifically affected by CHL, whereas the influence of mRNA splicing, maturation of SSU-rRNA and spliceosomal complex are only detected in THI treatment. More importantly, many functions are collectively disrupted by all three compounds, including extracellular exosome, oxidation-reduction process and drug metabolism - cytochrome P450, etc. This finding suggests the three compounds may share partly common mechanisms to induce cytotoxicity in liver. Moreover, we further tried to figure out the detailed molecular interaction implicated in those common mechanisms. The results show that several RNAs interactions (including mRNA-mRNA, lncRNA-mRNA) which involve 3 lncRNAs and 34 mRNAs conservatively existing in the transcriptome of damaged rat liver induced by the three compounds (Fig. 6-A). Particularly, three lncRNAs are found to interact significantly with an mRNA transcribed from MAO-b gene which is enriched in extracellular exosome and oxidation-reduction process. These three lncRNAs consist of 2 novel (MSTRG.23113.1, MSTRG.12375.1) and 1 known (ENSRNOT00000091624) transcript. MSTRG.12375.1 is found to be up-regulated in CHL group and ENSRNOT00000091624 is found to be commonly down-regulated in the three groups. Notably, their target mRNA ENSRNOT00000044009 transcribed from MAO-b exhibits to be commonly down-regulated in the three groups, which implies lncRNA MSTRG.12375.1 might negatively regulate its expression while lncRNA ENSRNOT00000091624 probably acts as enhancer for MAO-b. Also, our network analysis shows that MAO-b could interact with Gsta3 via Abcb1, Anxa7 and Pm20d1 (Fig. 6-B). Our functional analysis shows that Gsta3 is enriched in the GO function of chemical carcinogenesis. Indeed, Gsta3, which has already been proven to be involved in cellular defense against toxic and carcinogenic compounds, is found to be overexpressed in our study. Interestingly, Abcb1, Anxa7 and Pm20d1 are also up-regulated in the three groups. This finding might suggest that these lncRNAs indirectly participate in the process of chemical carcinogenesis guided by Gsta3 via MAO-b. Meanwhile, it has been illustrated that MAO-b, one of the AOs which are the major enzymes of BA metabolism, appear to be involved in programmed cell death 13, 14 and the manipulation of AOs could be considered as a mean of regulating tumor growth or inhibition. Another important finding is about glucuronidation. Related study showed that acyl-glucuronides induce inflammatory toxicity and cytotoxicity against CD14+ cells via the p38 mitogen-activated protein kinase pathway^15^. It is assumed that the chemical pathways of activation like glucuronidation initiate either direct or immune-mediated indirect hepatotoxicity^16^. The gene Ugt1a7 has been demonstrated to play an important role in glucuronidation activity toward benzo(a)pyrene phenol and diol metabolites^17^. Similarly, our network analysis shows that MAO-b could interact with Ugt1a7 through Kynu gene or through Npm1 gene and Rpl5 gene, which implies that the three lncRNAs might participate in the regulation of glucuronidation via MAO-b (Fig. 6-C). Regarding the functions of EE and ORP, they are two common metabolism processes in liver. According to the constructed network, the MAO-b gene could directly interact with Bbox1 or indirectly interact with Impdh2 through Ust5r. PPAR-induced FA oxidation has previously been associated with increased L-carnitine and acetylcarnitine in plasma^18^, ^19^, and with increased mRNA level of Bbox1 and Slc22a5^18^, ^20^, while in this study, hepatic Bbox1 mRNA, plasma L-carnitine and acetylcarnitine were lowered^21^. In our study, lipid peroxidation also exists as one of the injuries caused by reactive oxygen species derived from the metabolism of chemicals in liver. Similarly, mRNA of Bbox1 which directly interacts with the down-regulated mRNA of MAO-b gene is also down-regulated. There exists another interaction relationship between MAO-b and Impdh2 with common up-regulation in the three groups via Ust5r, but the mRNA of Ust5r is partially down-regulated (Fig. 6-D). Impdh2 is a kind of inosine monophosphate dehydrogenase which is strongly related to oxidation-reduction process. It is also up-regulated significantly in our study.

In summary, our study provides a global view for the complex RNA-RNA interactions (mRNA- mRNA and lncRNA-mRNA) in cytotoxicity-induced rat liver by constructing co-expression network, and we identified some candidate interactions in three aspects, including chemical carcinogenesis, glucuronidation and EE together with ORP. All these interactions are based on three lncRNAs containing 1 known transcript commonly down-regulated in the three groups and 2 novel transcripts with 1 of them partially up-regulated in the three groups. This implies some basic mechanism in some processes of pathogenic cytotoxicity. The results will improve our understanding of molecular mechanisms underlying the response to pathogenic cytotoxicity from complex co-expression network and help us discover true biomarkers for cytotoxic damaging.

## Methods and Materials

### Sampling and RNA sequencing

Our study was performed as a secondary analysis based on Wang Charles's experimental data^22^. The male Sprague-Dawley rats in the experiment came from Charles River laboratory. Three chemicals were used to induce cytotoxic damages to the liver. The chemicals were orally administered (10 ml/kg body weight in corn oil or water) or intraperitoneally, intravenously or subcutaneously injected (5 ml/kg body weight in saline). Raw RNA-Seq data was downloaded from the NCBI Gene Expression Omnibus (GEO) database with accession number GSE55347. Three samples were treated with carbon trichloride, two samples with carbon tetrachloride, and 12 control samples for both treatment groups. Three samples were treated with thioacetamide with five control samples.

### Data processing, alignment and assembly

Firstly, Trimmomatic (version 0.36)^23^ was used to filter paired-end reads of each sample, eliminating adapter and low-quality reads (ILLUMINACLIP: TruSeq3-PE.fa:2:30:10:8:true SLIDINGWINDOW:4:15 LEADING:3 TRAILING:3 MINLEN:50). Then the clean reads were aligned to the *Rattus norvegicus* genome sequences (Ensembl release 92) using STAR in 2-pass mode^24^. Finally, StringTie^25^ was used to assemble the transcripts.

### Differential expression analysis

We used featurecounts (v1.5.3)^26^ to quantify the transcripts, then used R package DESeq2^27^ to normalize the counts of mapped reads and to analyze the differential expression of transcripts. Finally, we applied Benjamini-Hochberg method and screened the transcripts with FDR ≤0.05.

### Identification of lncRNAs

We proposed a rigorous filtering pipeline (Fig. 1) to identify lncRNA. Firstly, 'biotype transcript', which belongs to lncRNA, was extracted from the assembled transcript annotation file. The marked mRNA was extracted in the same way. Then the remaining transcripts were queried in Uniprot database, NCBI non-redundant protein database, and protein sequences dataset of *R. norvegicus* derived from Ensembl database, sequences with hits in these databases were considered coding sequences. After removing these coding transcripts and filtering out the transcripts with length greater than 200 nt and longest ORF less than 100 amino acids, we used CPC and Pfam software to predict protein coding potential on the remaining transcripts. Removing the transcripts predicted to be encoding proteins, the remaining was considered lncRNAs.

### Modules identification using WGCNA analysis

Firstly, the control samples from all three treatment-control comparisons were merged, because it is commonly believed that different control methods in this experiment will not result in differential transcription in liver tissue. Then we extracted the normalized reads count based on regularized log normalization method in DESeq2. We used these normalized matrices to pick up the soft threshold value using WGCNA package. For carbon tetrachloride, the soft threshold we picked up is 4 after observing the normal result of the samples clustering (Fig. S6-A, B). Similarly, we got the soft thresholds 3 in THI (Fig. S6-E, F). In CHL, we removed the outlier sample SRR1177989 and SRR1178004 from the first sample clustering result, then got the soft threshold 5 (Fig. S6-C, D). Subsequently, we applied the best soft threshold value in each group to construct the co-expression network of gene. From each co-expression network, we identified some modules accordingly. Next, we need to find out the special modules which are strongly related to the three compounds. Hence, we designed three matrices which contain a column to differentiate the case subtypes. When a sample belongs to the control group, then set 0 as the subtype value, otherwise set as 1. Eigengenes were calculated to display the correlation between each module using the function *moduleEigengenes* in WGCNA package. Furthermore, the designed matrix in each group was added into this step to calculate the correlation between modules and subtypes. The most significant modules will be selected for downstream analysis (Fig. 5).

Subsequently, we extracted the gene IDs of the most significant module in each group as identifiers and used the gene IDs of all assembled genes as background to perform functional enrichment analysis with DAVID. The statistically significant GO categories and KEGG pathways with an EASE score (modified Fisher Exact P-Value) ≤ 0.05 were obtained using the Fisher's Exact test (Fig. S12-S23).

From the result of functional enrichment analysis, the overlapping GO categories and KEGG pathways will be retained, and their common genes in each term can also be identified (Table. 1). These genes are considered as key elements involved in the enriched terms.

### Identification of pairs of interactions between the RNAs (including mRNA-mRNA and mRNA-lncRNA)

From the most significant modules which are related to the properties identified in each group, we can calculate TOM similarity using the function *TOMsimilarityFromExpr* in WGCNA package with the best soft threshold value. The TOM similarity matrices are potential interactions between all RNAs with a weighted value. According to the TOM similarity matrices, we obtained the interactions existed in common in three groups. Moreover, we picked out the mRNA-mRNA and mRNA-lncRNA interactions related to the common genes found in enriched GO terms, each of which is enriched GO terms and pathways commonly found in all three groups. To get more reliable interactions, we only extracted top 20% of the common pairs by the weighted values in each group (Fig. 6).

## Supplementary Material

Supplementary figures and table S1-3.

Supplementary table S4.

Supplementary table S5.

## Figures and Tables

**Table 1:** Function enrichment common in three groups

Number	Category	Term	Common gene number
1	GOTERM_CC_DIRECT	GO:0070062~extracellular exosome	36
2	GOTERM_BP_DIRECT	GO:0052697~xenobiotic glucuronidation	8
3	GOTERM_BP_DIRECT	GO:0052696~flavonoid glucuronidation	8
4	KEGG_PATHWAY	rno00982: Drug metabolism - cytochrome P450	11
5	KEGG_PATHWAY	rno00053: Ascorbate and aldarate metabolism	9
6	GOTERM_BP_DIRECT	GO:0009813~flavonoid biosynthetic process	7
7	GOTERM_MF_DIRECT	GO:0015020~glucuronosyltransferase activity	8
8	KEGG_PATHWAY	rno00983: Drug metabolism - other enzymes	9
9	GOTERM_BP_DIRECT	GO:0006412~translation	14
10	KEGG_PATHWAY	rno00040: Pentose and glucuronate interconversions	8
11	KEGG_PATHWAY	rno05204: Chemical carcinogenesis	10
12	GOTERM_BP_DIRECT	GO:0052695~cellular glucuronidation	4
13	KEGG_PATHWAY	rno00860: Porphyrin and chlorophyll metabolism	8
14	KEGG_PATHWAY	rno01100: Metabolic pathways	21
15	GOTERM_BP_DIRECT	GO:0001889~liver development	6
16	GOTERM_BP_DIRECT	GO:0055114~oxidation-reduction process	9
17	KEGG_PATHWAY	rno00980: Metabolism of xenobiotics by cytochrome P450	11

## Abbreviations

ROS: reactive oxygen species
CAR: carbon tetrachloride
CHL: chloroform
THI: thioacetamide
CPC: Coding Potential Calculator
SDE: significantly differentially expressed
PCC: Pearson Correlation Coefficient
TOM: Topology Overlap Matrix
AOs: amine oxidases
BA: biogenic amine
EE: extracellular exosome
ORP: oxidation-reduction process
FA: fatty acid
FDR: false discovery rate
WGCNA: weighted correlation network analysis

## References

[1] QuickStats (2017). Death Rates* for Chronic Liver Disease and Cirrhosis,(dagger) by Sex and Age Group - National Vital Statistics System, United States, 2000 and 2015. MMWR Morb Mortal Wkly Rep.

[2] Devasagayam TP, Tilak JC, Boloor KK, Sane KS, Ghaskadbi SS, Lele RD (2004). Free radicals and antioxidants in human health: current status and future prospects. J Assoc Physicians India.

[3] Ruch RJ, Klaunig JE, Schultz NE, Askari AB, Lacher DA, Pereira MA (1986). Mechanisms of chloroform and carbon tetrachloride toxicity in primary cultured mouse hepatocytes. Environ Health Perspect.

[4] Boll M, Weber LW, Becker E, Stampfl A (2001). Mechanism of carbon tetrachloride-induced hepatotoxicity. Hepatocellular damage by reactive carbon tetrachloride metabolites. Z Naturforsch C.

[5] Low TY, Leow CK, Salto-Tellez M, Chung MC (2004). A proteomic analysis of thioacetamide-induced hepatotoxicity and cirrhosis in rat livers. Proteomics.

[6] Wilusz JE, Sunwoo H, Spector DL (2009). Long noncoding RNAs: functional surprises from the RNA world. Genes Dev.

[7] Zhao Y, Wu J, Liangpunsakul S, Wang L (2017). Long Non-coding RNA in Liver Metabolism and Disease: Current Status. Liver Res.

[8] Smekalova EM, Kotelevtsev YV, Leboeuf D, Shcherbinina EY, Fefilova AS, Zatsepin TS (2016). lncRNA in the liver: Prospects for fundamental research and therapy by RNA interference. Biochimie.

[9] Atanasovska B, Rensen SS, van der Sijde MR, Marsman G, Kumar V, Jonkers I (2017). A liver-specific long noncoding RNA with a role in cell viability is elevated in human nonalcoholic steatohepatitis. Hepatology.

[10] Li P, Ruan X, Yang L, Kiesewetter K, Zhao Y, Luo H (2015). A liver-enriched long non-coding RNA, lncLSTR, regulates systemic lipid metabolism in mice. Cell Metab.

[11] Fan YH, Wu MJ, Jiang Y, Ye M, Lu SG, Wu L (2017). Long non-coding RNA HULC as a potential prognostic biomarker in human cancers: a meta-analysis. Oncotarget.

[12] Lan X, Yan J, Ren J, Zhong B, Li J, Li Y (2016). A novel long noncoding RNA Lnc-HC binds hnRNPA2B1 to regulate expressions of Cyp7a1 and Abca1 in hepatocytic cholesterol metabolism. Hepatology.

[13] Hangxiu X, Rupesh C, Yulan C, Bussiere FI, Mohammad A, Yao MD (2004). Spermine oxidation induced by Helicobacter pylori results in apoptosis and DNA damage: implications for gastric carcinogenesis. Cancer Research.

[14] Kunduzova OR, Bianchi P, Pizzinat N, Escourrou G, Seguelas MH, Parini A (2002). Regulation of JNK/ERK activation, cell apoptosis, and tissue regeneration by monoamine oxidases after renal ischemia-reperfusion. Faseb Journal Official Publication of the Federation of American Societies for Experimental Biology.

[15] Miyashita T, Kimura K, Fukami T, Nakajima M, Yokoi T (2014). Evaluation and mechanistic analysis of the cytotoxicity of the acyl glucuronide of nonsteroidal anti-inflammatory drugs. Drug Metab Dispos.

[16] Spahnlangguth H, Benet LZ (1992). Acyl glucuronides revisited: is the glucuronidation process a toxification as well as a detoxification mechanism?. Drug Metabolism Reviews.

[17] Grove AD, Kessler FK, Metz RP, Ritter JK (1997). Identification of a rat oltipraz-inducible UDP-glucuronosyltransferase (UGT1A7) with activity towards benzo(a)pyrene-7,8-dihydrodiol. Journal of Biological Chemistry.

[18] Burri L, Thoresen GH, Berge RK (2010). The Role of PPARα Activation in Liver and Muscle. PPAR Research,2010,(2010-07-28).

[19] Burri L, Bjørndal B, Wergedahl H, Berge K, Bohov P, Svardal A (2011). Tetradecylthioacetic Acid Increases Hepatic Mitochondrial β-Oxidation and Alters Fatty Acid Composition in a Mouse Model of Chronic Inflammation. Lipids.

[20] Makowski L, Noland RC, Koves TR, Xing WB, Ilkayeva OR, Muehlbauer MJ (2009). Metabolic profiling of PPARα-/- mice reveals defects in carnitine and amino acid homeostasis that are partially reversed by oral carnitine supplementation. Faseb Journal.

[21] Lindquist C, Bjørndal B, Rossmann CR, Tusubira D, Svardal A, Røsland GV (2017). Increased Hepatic Mitochondrial FA Oxidation leads to lower TG levels in Rat Liver and Plasma, and is associated with Upregulation of Uncoupling Proteins and Downregulation of Apolipoprotein C-III.

[22] Wang C, Gong B, Bushel PR, Thierry-Mieg J, Thierry-Mieg D, Xu J (2014). The concordance between RNA-seq and microarray data depends on chemical treatment and transcript abundance. Nat Biotechnol.

[23] Bolger AM, Lohse M, Usadel B (2014). Trimmomatic: a flexible trimmer for Illumina sequence data. Bioinformatics.

[24] Dobin A, Davis CA, Schlesinger F, Drenkow J, Zaleski C, Jha S (2013). STAR: ultrafast universal RNA-seq aligner. Bioinformatics.

[25] Pertea M, Pertea GM, Antonescu CM, Chang TC, Mendell JT, Salzberg SL (2015). StringTie enables improved reconstruction of a transcriptome from RNA-seq reads. Nat Biotechnol.

[26] Liao Y, Smyth GK, Shi W (2014). featureCounts: an efficient general purpose program for assigning sequence reads to genomic features. Bioinformatics.

[27] Love MI, Huber W, Anders S (2014). Moderated estimation of fold change and dispersion for RNA-seq data with DESeq2. Genome Biol.

