# Supplementary material for: Co-expression network analysis of lncRNAs and mRNAs in rat liver tissue reveals the complex interactions in response to pathogenic cytotoxicity
Source: Int J Biol Sci. 2019 Aug 22;15(11):2296–307. doi: 10.7150/ijbs.33735 (PMC6775316; doi:10.7150/ijbs.33735)

Content

Table S1. Basic statistics of deep RNA sequencing data before and after processing. 1

Table S2. Basic statistics of clean reads alignment against to the *Rattus norvegicus* genome sequence. 2

Table S3. Basic statistics of assembly results of transcriptome in *Rattus norvegicus*. 3

Figure S1: Distribution of five different types of alternative splicing events identified in three chemical treatment. 4

Figure S2: Distribution of the lncRNAs identified in three chemical treatment. 5

Figure S3: Length distribution of all assembled known and novel lncRNAs from *Rattus norvegicus* transcriptome. 6

Figure S4: PCA analysis for the samples in three chemical treatment. 7

Figure S5: Samlples clustering to detect outliers for three group. 8

Figure S6: Identification of best soft threshold among three groups. 9

Figure S7: Cluster dendrogram among three groups. 10

Figure S8: Module membership vs. gene significance among three groups. 11

Figure S9: Eigengene adjacency heatmap illustrating the relationship between modules and traits in CAR group. 12

Figure S10: Eigengene adjacency heatmap illustrating the relationship between modules and traits in CHL group. 12

Figure S11: Eigengene adjacency heatmap illustrating the relationship between modules and traits in THI group. 14

Figure S12: Functional enrichment analysis for biological process after CAR chemical treatment using DAVID server. 15

Figure S13: Functional enrichment analysis for cellular component after CAR chemical treatment using DAVID server. 16

Figure S14: Functional enrichment analysis for molecular function after CAR chemical treatment using DAVID server. 17

Figure S15: KEGG analysis for molecular function after CAR chemical treatment using DAVID server. 18

Figure S16: Functional enrichment analysis for biological process after CHL chemical treatment using DAVID server. 19

Figure S17: Functional enrichment analysis for cellular component after CHL chemical treatment using DAVID server. 20

Figure S18: Functional enrichment analysis for molecular function after CHL chemical treatment using DAVID server. 21

Figure S19: KEGG analysis for molecular function after CHL chemical treatment using DAVID server. 22

Figure S20: Functional enrichment analysis for biological process after THI chemical treatment using DAVID server. 23

Figure S21: Functional enrichment analysis for cellular component after THI chemical treatment using DAVID server. 24

Figure S22: Functional enrichment analysis for molecular function after THI chemical treatment using DAVID server. 25

Figure S23: KEGG analysis for molecular function after THI chemical treatment using DAVID server. 26

Table S1. Basic statistics of deep RNA sequencing data before and after processing.

Sample ID	group	Status[a]	chemical	Raw reads	Raw base	Clean reads	Clean base
SRR1177973	control	control_1,2_rep1	none	31,177,368	3,148,914,168	25,432,020	2,462,728,432
SRR1178016	control	control_1,2_rep2	none	37,633,288	1,881,664,400	30,379,728	1,518,986,400
SRR1178019	control	control_1,2_rep3	none	31,890,846	3,189,084,600	24,346,292	2,193,935,958
SRR1178024	control	control_1,2_rep4	none	34,863,516	3,486,351,600	26,738,504	2,413,857,762
SRR1178035	control	control_1,2_rep5	none	28,881,662	2,888,166,200	12,219,280	1,024,689,960
SRR1178045	control	control_1,2_rep6	none	43,991,002	4,399,100,200	37,598,592	3,476,914,366
SRR1178050	control	control_1,2_rep7	none	32,173,238	3,217,323,800	26,457,064	2,469,251,284
SRR1178061	control	control_1,2_rep8	none	126,534,710	12,780,005,710	113,578,792	11,094,937,146
SRR1178063	control	control_1,2_rep9	none	89,067,134	8,995,780,534	78,194,796	7,630,920,826
SRR1178064	control	control_1,2_rep10	none	114,879,700	11,602,849,700	103,271,474	10,094,821,394
SRR1178074	control	control_1,2_rep11	none	100,372,668	10,137,639,468	89,651,088	8,752,622,686
SRR1178075	control	control_1,2_rep12	none	84,037,926	8,487,830,526	71,991,346	7,031,898,580
SRR1177974	1	case_1_rep1	CARBON TETRACHLORIDE	37,111,098	3,748,220,898	28,934,614	2,782,585,288
SRR1177976	1	case_1_rep2	CARBON TETRACHLORIDE	32,472,640	3,279,736,640	25,072,632	2,400,528,770
SRR1177987	2	case_2_rep1	CHLOROFORM	27,286,434	2,622,275,456	27,286,434	2,615,452,952
SRR1177988	2	case_2_rep2	CHLOROFORM	28,309,072	2,702,468,469	28,309,072	2,700,210,018
SRR1177989	2	case_2_rep3	CHLOROFORM	29,236,332	2,830,795,061	29,236,332	2,813,330,092
SRR1178004	control	control_3_rep1	none	35,195,190	3,368,980,094	35,195,190	3,425,939,420
SRR1178006	control	control_3_rep2	none	38,334,282	3,678,690,904	38,334,282	3,733,890,438
SRR1178013	control	control_3_rep3	none	29,241,722	2,835,875,928	29,241,722	2,865,263,524
SRR1178062	control	control_3_rep4	none	113,793,168	11,185,355,795	113,793,168	11,116,172,178
SRR1178070	control	control_3_rep5	none	84,237,388	8,329,607,546	84,237,388	8,281,855,076
SRR1177966	3	case_3_rep1	THIOACETAMIDE	30,921,234	3,020,482,337	30,921,234	3,006,946,166
SRR1177969	3	case_3_rep2	THIOACETAMIDE	30,633,024	2,975,058,834	30,633,024	2,957,983,402
SRR1177970	3	case_3_rep3	THIOACETAMIDE	30,137,272	2,917,660,816	30,137,272	2,900,399,800

[a]. control_1,2_* means the common control for group 1 and group 2; control_3_* means the control for group 3.

Table S2. Basic statistics of clean reads alignment against to the *Rattus norvegicus* genome sequence.

Sample ID	group	Status[b]	chemical	Mapped reads	Mapped rate (%)
SRR1177973	control	control_1,2_rep1	none	22, 442, 236	88. 24
SRR1178016	control	control_1,2_rep2	none	26, 312, 986	86. 61
SRR1178019	control	control_1,2_rep3	none	21, 343, 476	87. 67
SRR1178024	control	control_1,2_rep4	none	23, 180, 590	86. 69
SRR1178035	control	control_1,2_rep5	none	10, 584, 642	86. 62
SRR1178045	control	control_1,2_rep6	none	32, 773, 378	87. 17
SRR1178050	control	control_1,2_rep7	none	23, 127, 204	87. 41
SRR1178061	control	control_1,2_rep8	none	100, 875, 084	88. 82
SRR1178063	control	control_1,2_rep9	none	70, 303, 084	89. 91
SRR1178064	control	control_1,2_rep10	none	91, 735, 658	88. 83
SRR1178074	control	control_1,2_rep11	none	78, 524, 370	87. 59
SRR1178075	control	control_1,2_rep12	none	64, 456, 258	89. 53
SRR1177974	1	case_1_rep1	CARBON TETRACHLORIDE	26, 068, 042	90. 09
SRR1177976	1	case_1_rep2	CARBON TETRACHLORIDE	22, 429, 210	89. 46
SRR1177987	2	case_2_rep1	CHLOROFORM	24, 486, 020	89. 74
SRR1177988	2	case_2_rep2	CHLOROFORM	25, 690, 438	90. 75
SRR1177989	2	case_2_rep3	CHLOROFORM	26, 166, 592	89. 50
SRR1178004	control	control_3_rep1	none	30, 481, 220	86. 61
SRR1178006	control	control_3_rep2	none	33, 915, 942	88. 47
SRR1178013	control	control_3_rep3	none	26, 203, 538	89. 61
SRR1178062	control	control_3_rep4	none	100, 138, 330	88. 00
SRR1178070	control	control_3_rep5	none	75, 805, 910	89. 99
SRR1177966	3	case_3_rep1	THIOACETAMIDE	27, 590, 188	89. 23
SRR1177969	3	case_3_rep2	THIOACETAMIDE	27, 662, 128	90. 30
SRR1177970	3	case_3_rep3	THIOACETAMIDE	27, 204, 158	90. 27

[b.] control_1,2_* means the common control for group 1 and group 2; control_3_* means the control for group 3.

Table S3. Basic statistics of assembly results of transcriptome in *Rattus norvegicus*.

Statistics terms	Number
Total number	64,938
Total length of (bp)	121,882,090
Average length (bp)	1,876.90
N50 Length (bp)	3,588
Maximum length (bp)	41,083
Minimum length (bp)	26
GC content (%)	48.76

Figure S1: Distribution of five different types of alternative splicing events identified in three chemical treatment.

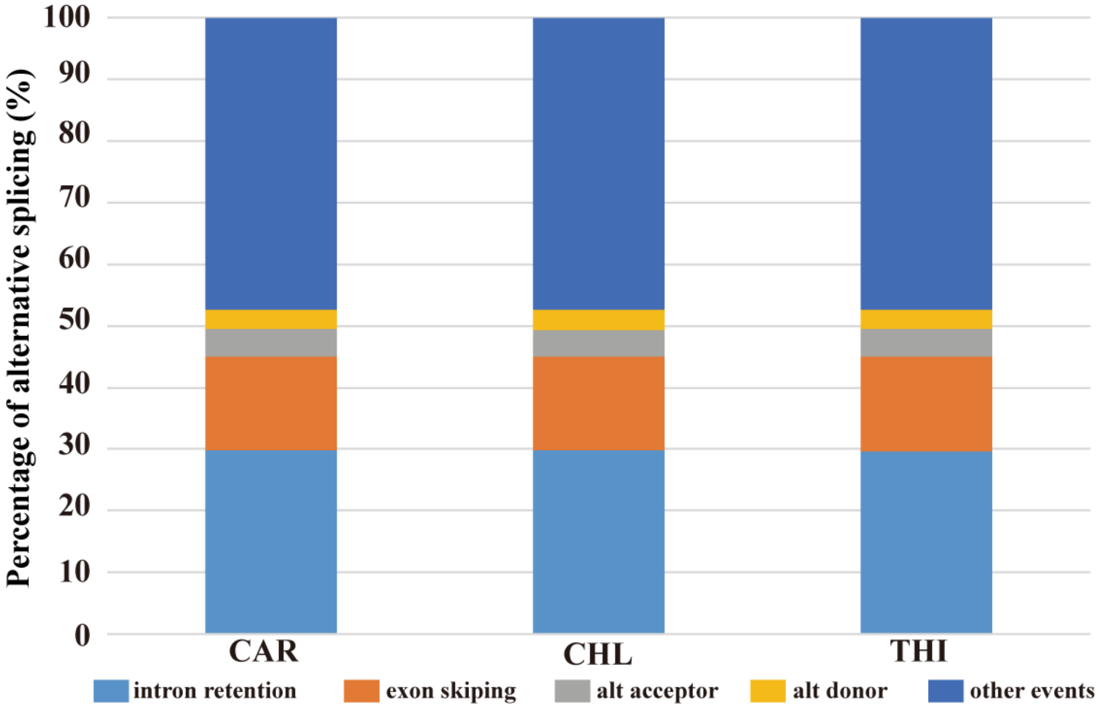

Figure S2: Distribution of the lncRNAs identified in three chemical treatment.

(A) Distribution of the known lncRNAs identified in three chemical treatment. (B) Distribution of the novel lncRNAs identified in three chemical treatment.

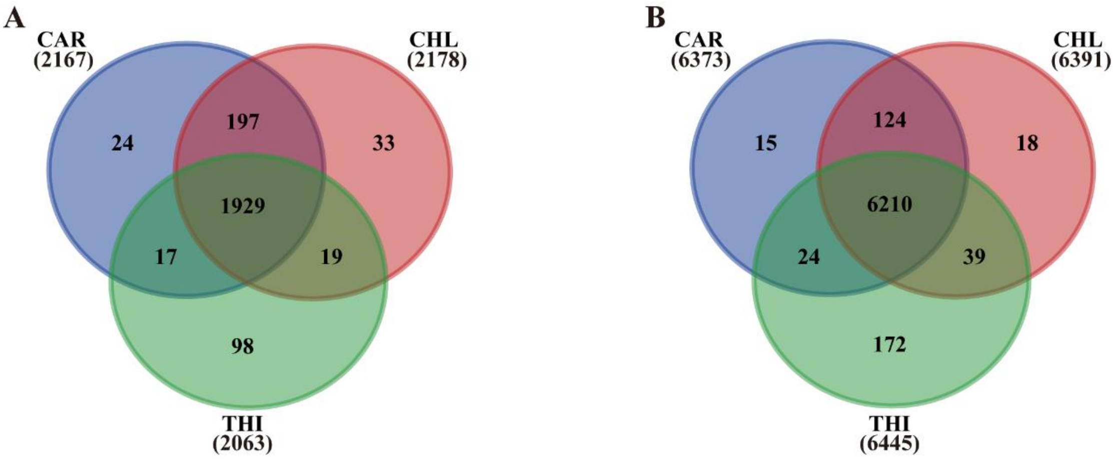

Figure S3: Length distribution of all assembled known and novel lncRNAs from *Rattus norvegicus* transcriptome.

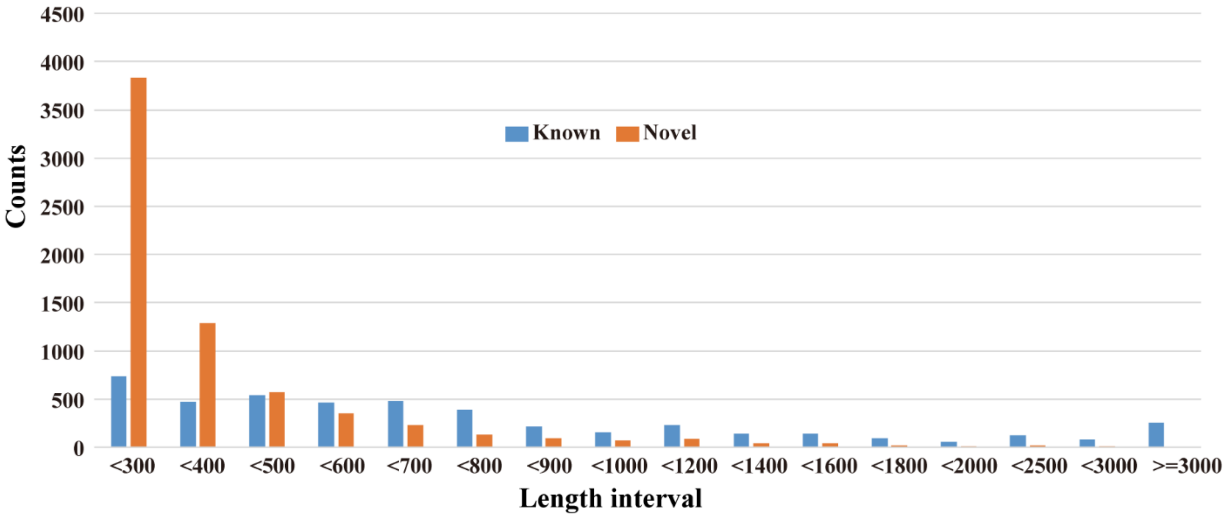

Figure S4: PCA analysis for the samples in three chemical treatment.

(A) PCA analysis for the samples in CAR chemical treatment. (B) PCA analysis for the samples in CHL chemical treatment. (C) PCA analysis for the samples in THI chemical treatment.

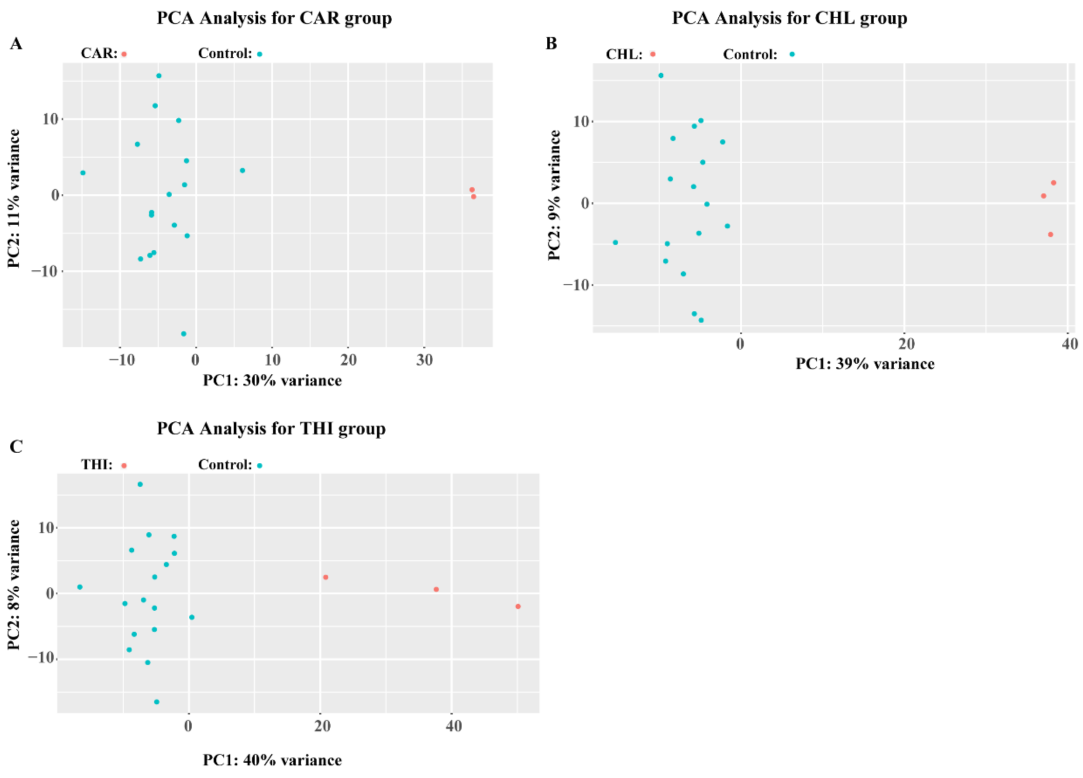

Figure S5: Samlples clustering to detect outliers for three group.

(A) Samlples clustering to detect outliers for CAR group. (B) Samlples clustering to detect outliers for CHL group. (C) Samlples clustering for CHL group after removing the outliers. (D) Samlples clustering to detect outliers for THI group.

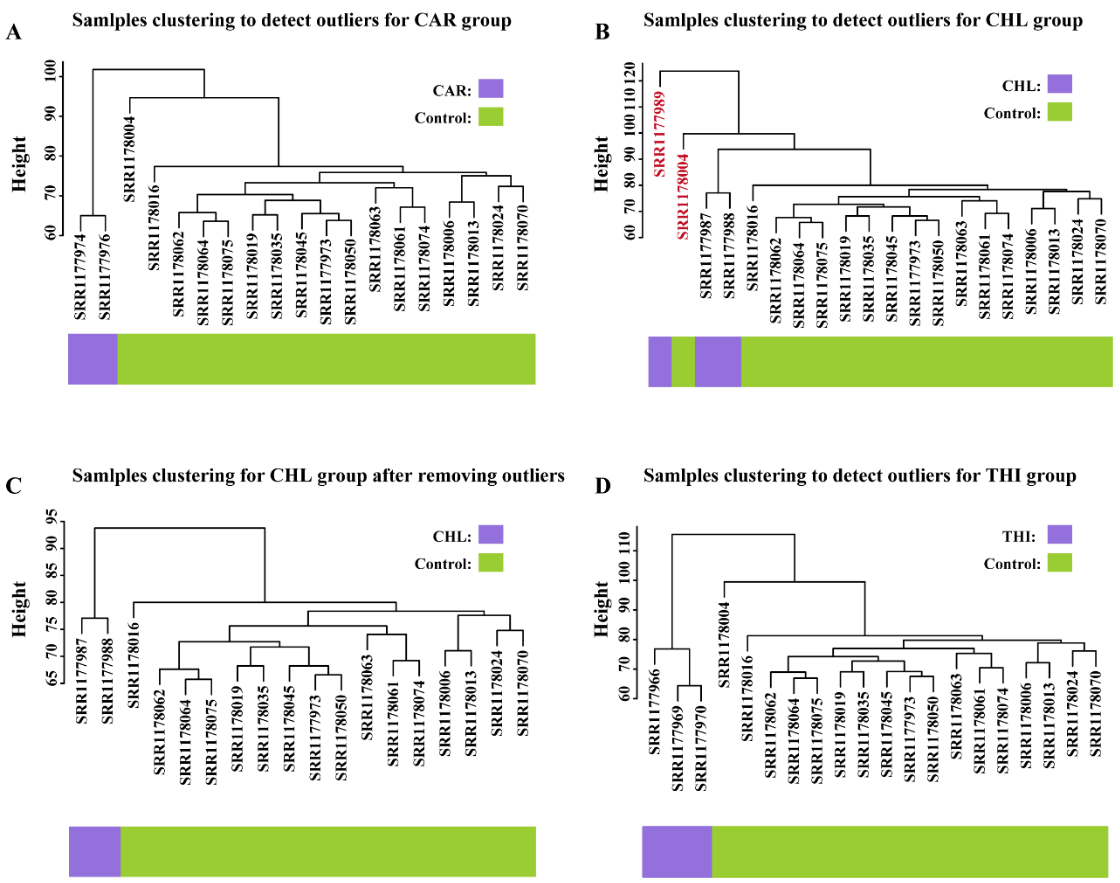

Figure S6: Identification of best soft threshold among three groups.

(A) Scale independence in CAR group. (B) Mean connectivity in CAR group. (C) Scale independence in CHL group. (D) Mean connectivity in CHL group. (E) Scale independence in THI group. (F) Mean connectivity in THI group.

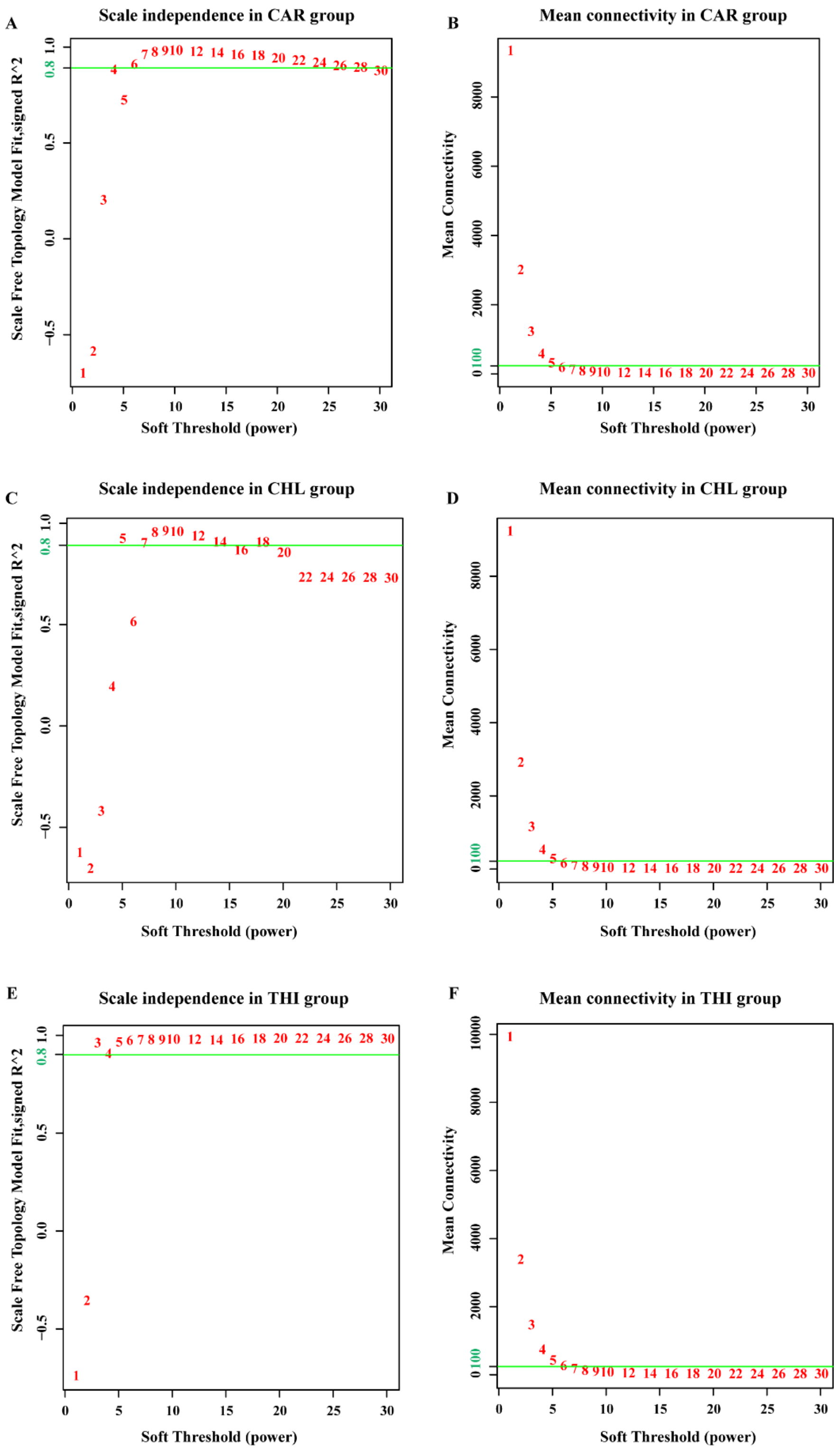

Figure S7: Cluster dendrogram among three groups.

(A) Cluster dendrogram among CAR groups. (B) Cluster dendrogram among CHL groups. (C) Cluster dendrogram among THI groups.

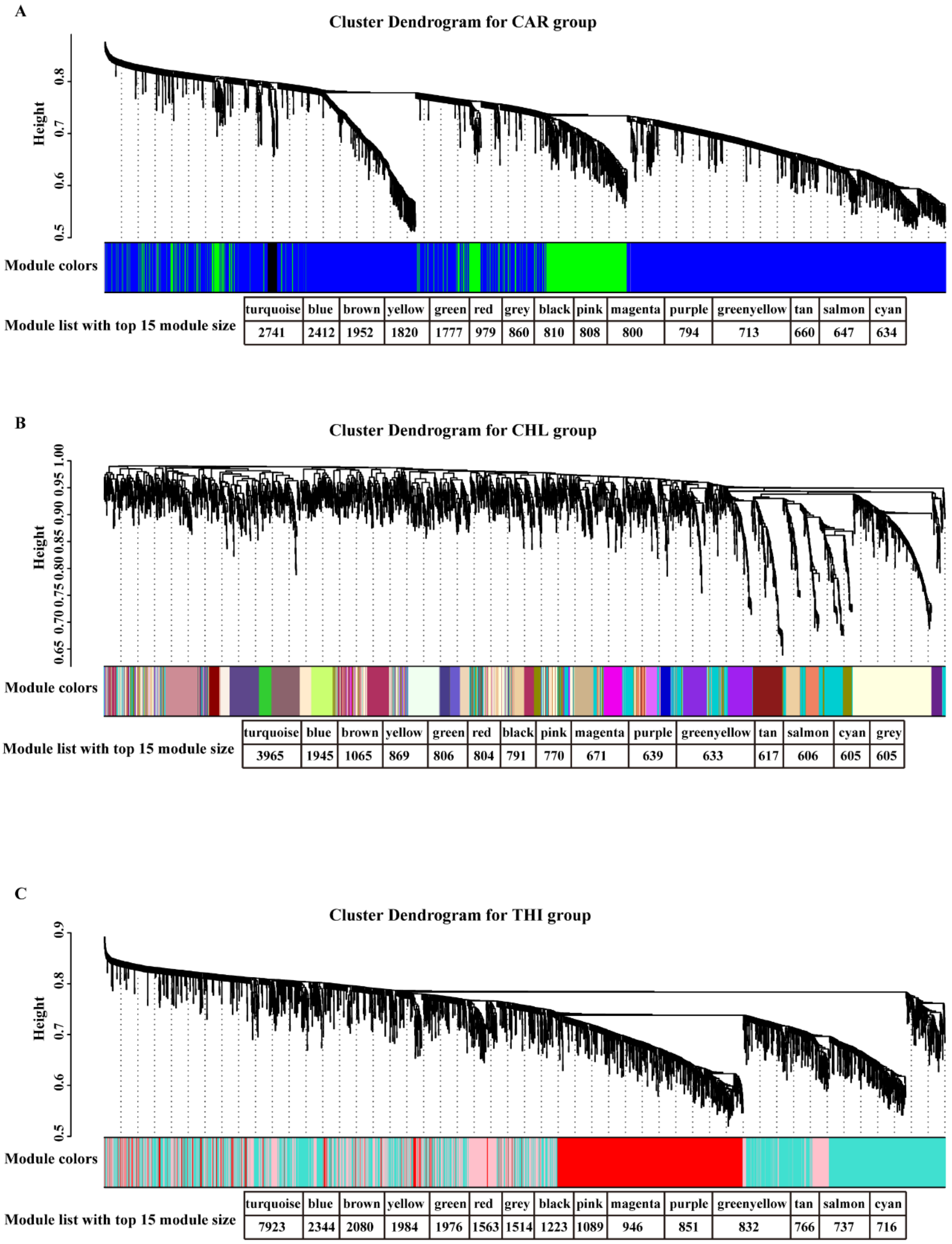

A — Module list with top 15 module size

turquoise	blue	brown	yellow	green	red	grey	black	pink	magenta	purple	greenyellow	tan	salmon	cyan
2741	2412	1952	1820	1777	979	860	810	808	800	794	713	660	647	634

B — Module list with top 15 module size

turquoise	blue	brown	yellow	green	red	black	pink	magenta	purple	greenyellow	tan	salmon	cyan	grey
3965	1945	1065	869	806	804	791	770	671	639	633	617	606	605	605

C — Module list with top 15 module size

turquoise	blue	brown	yellow	green	red	grey	black	pink	magenta	purple	greenyellow	tan	salmon	cyan
7923	2344	2080	1984	1976	1563	1514	1223	1089	946	851	832	766	737	716

Figure S8: Module membership vs. gene significance among three groups.

(A) Module membership vs. gene significance among CAR groups. (B) Module membership vs. gene significance among CHL groups. (C) Module membership vs. gene significance THI three groups.

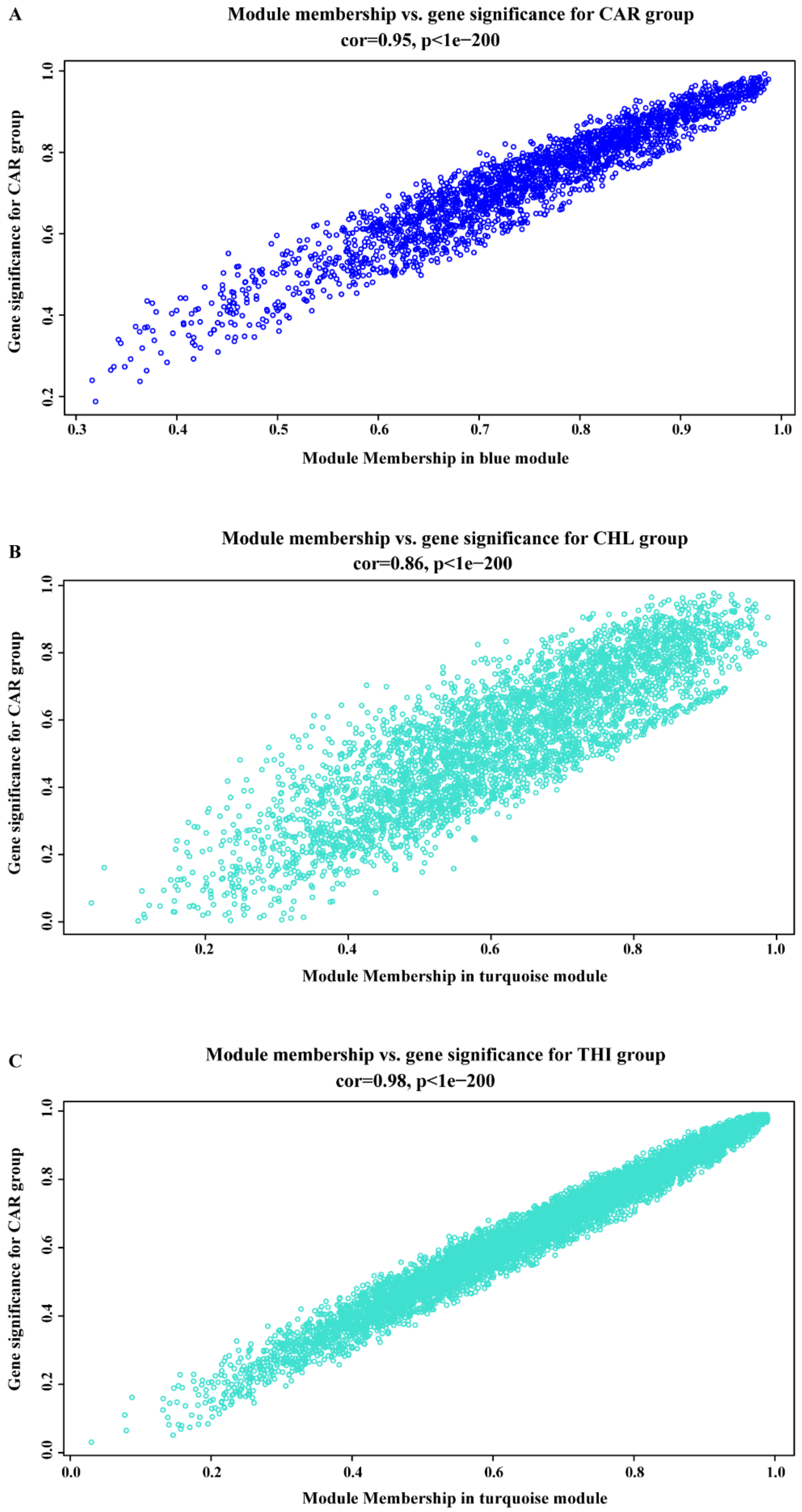

Figure S9: Eigengene adjacency heatmap illustrating the relationship between modules and traits in CAR group.

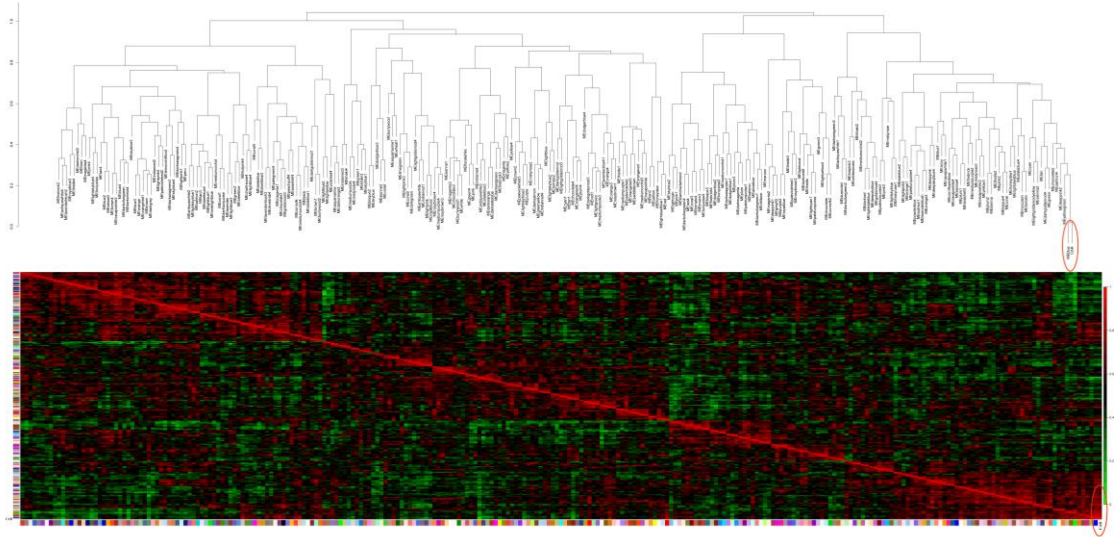

Figure S10: Eigengene adjacency heatmap illustrating the relationship between modules and traits in CHL group.

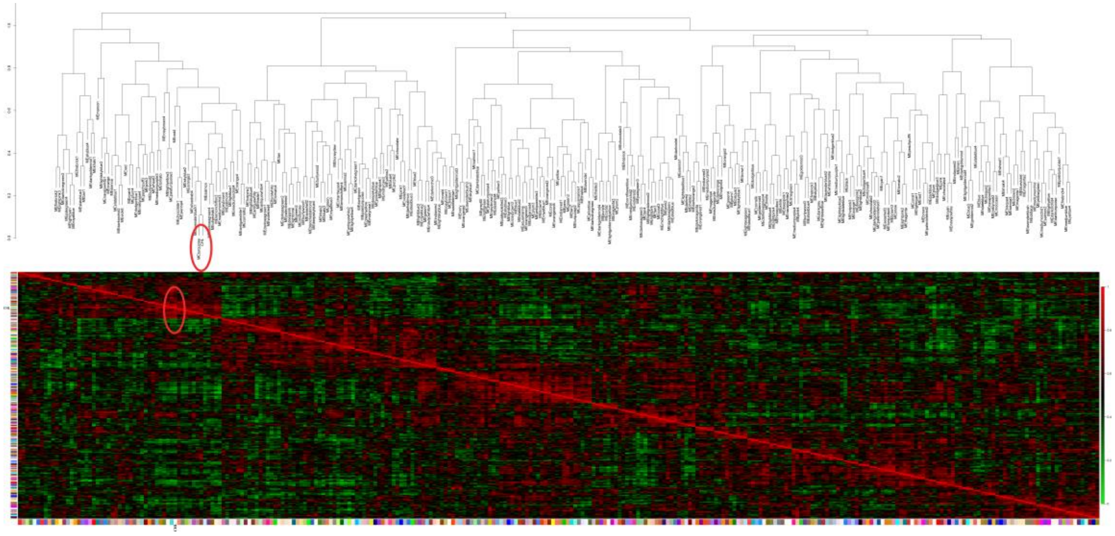

Figure S11: Eigengene adjacency heatmap illustrating the relationship between modules and traits in THI group.

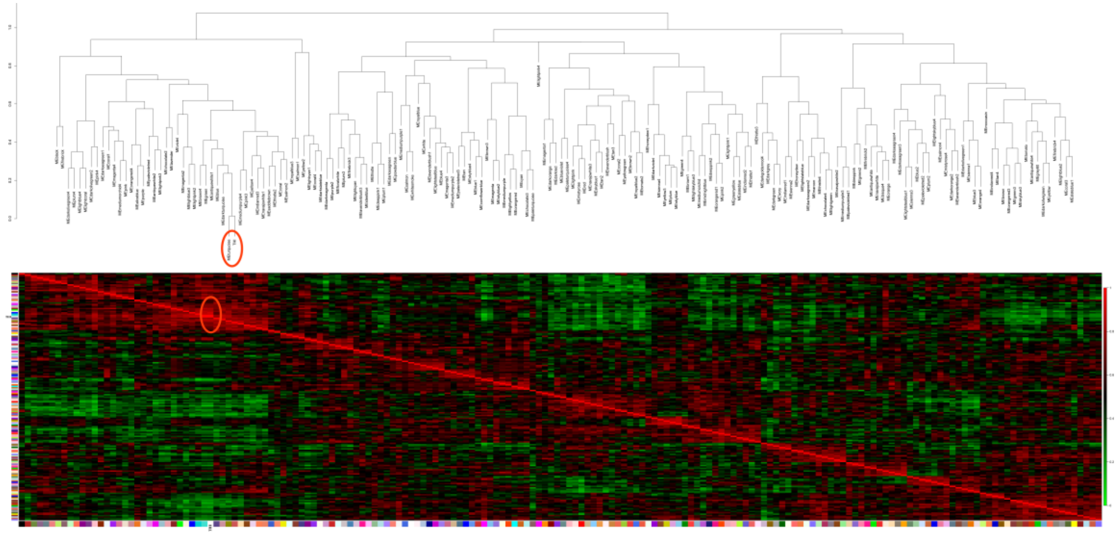

Figure S12: Functional enrichment analysis for biological process after CAR chemical treatment using DAVID server.

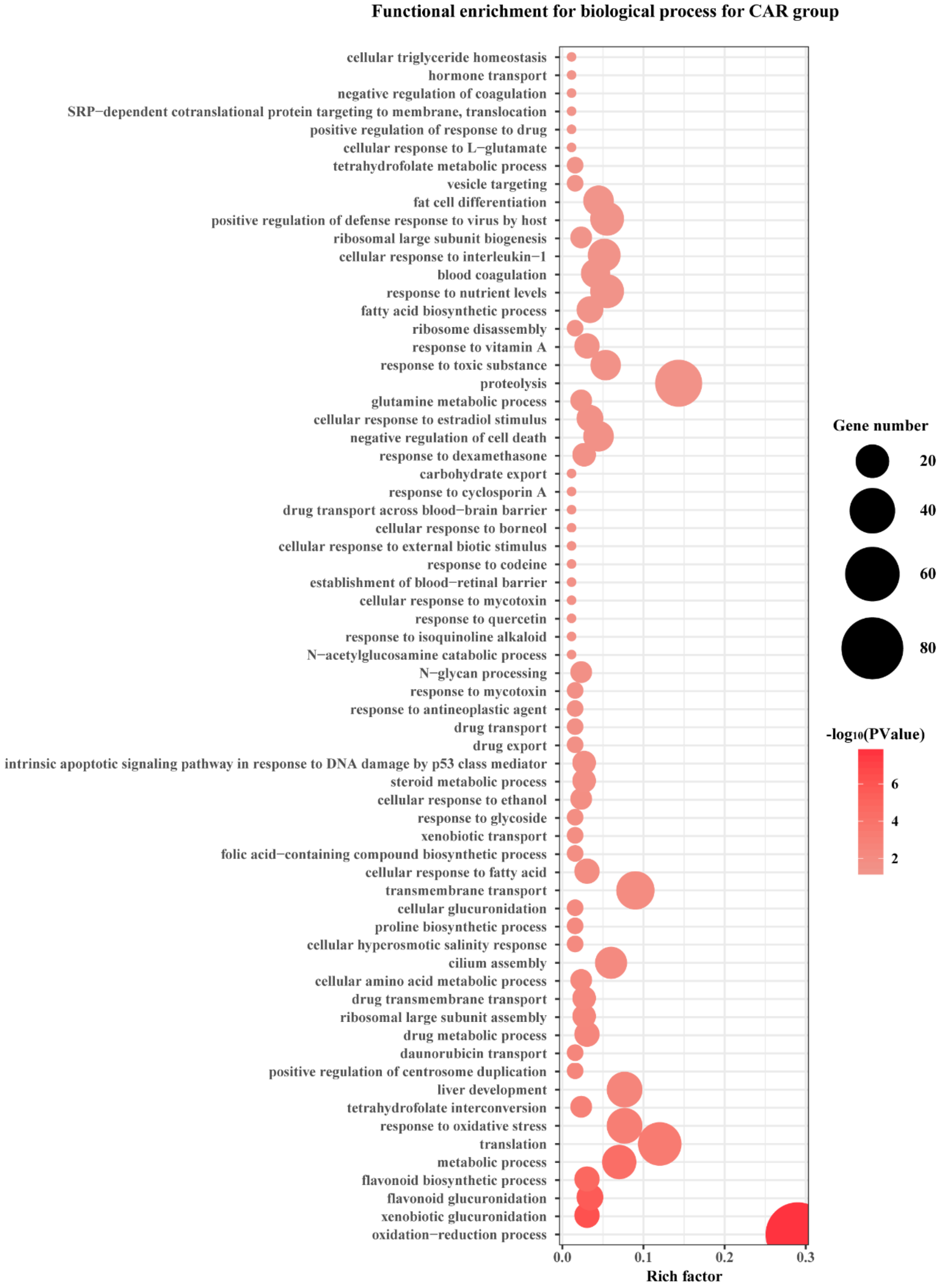

Figure S13: Functional enrichment analysis for cellular component after CAR chemical treatment using DAVID server.

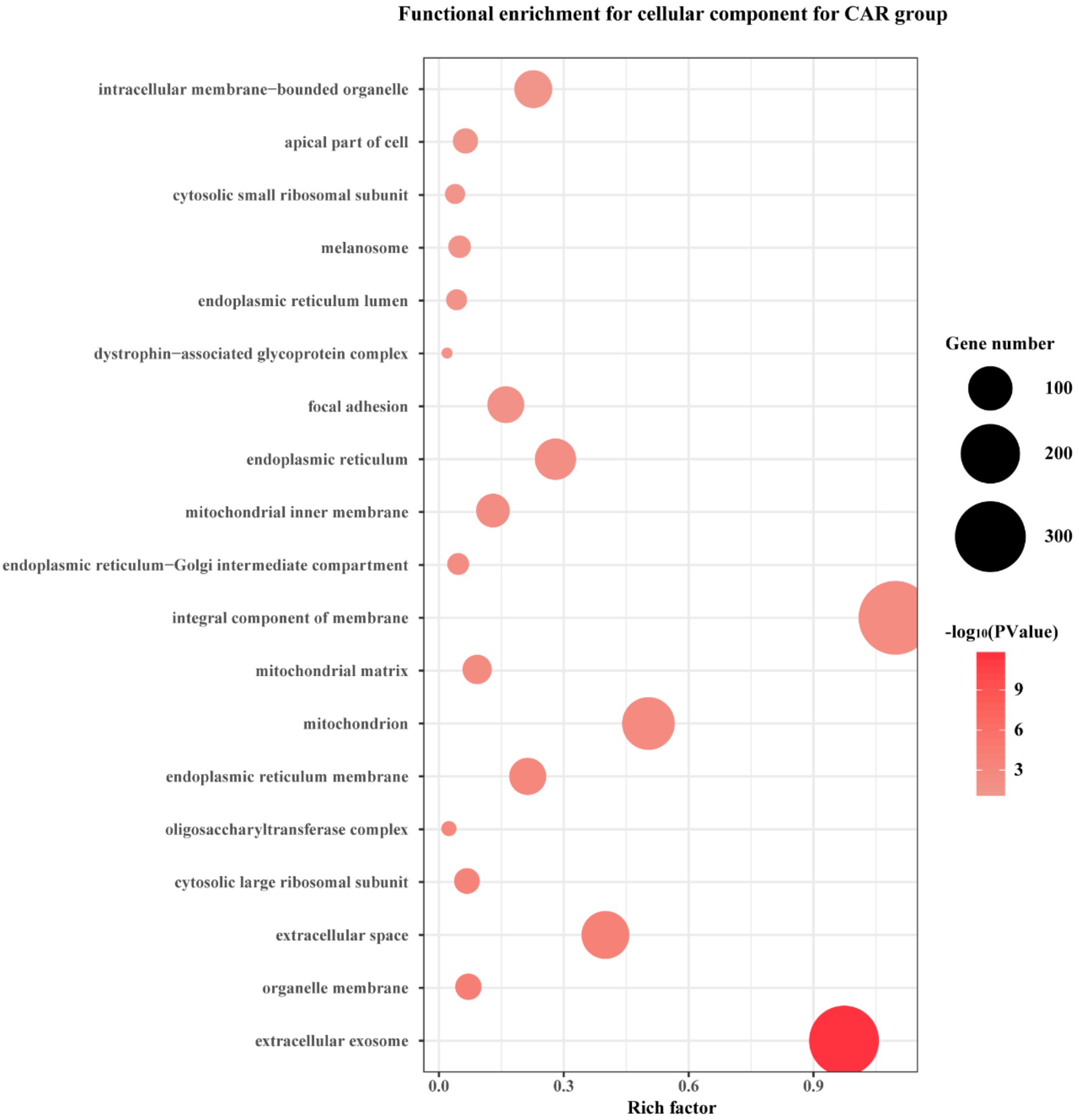

Figure S14: Functional enrichment analysis for molecular function after CAR chemical treatment using DAVID server.

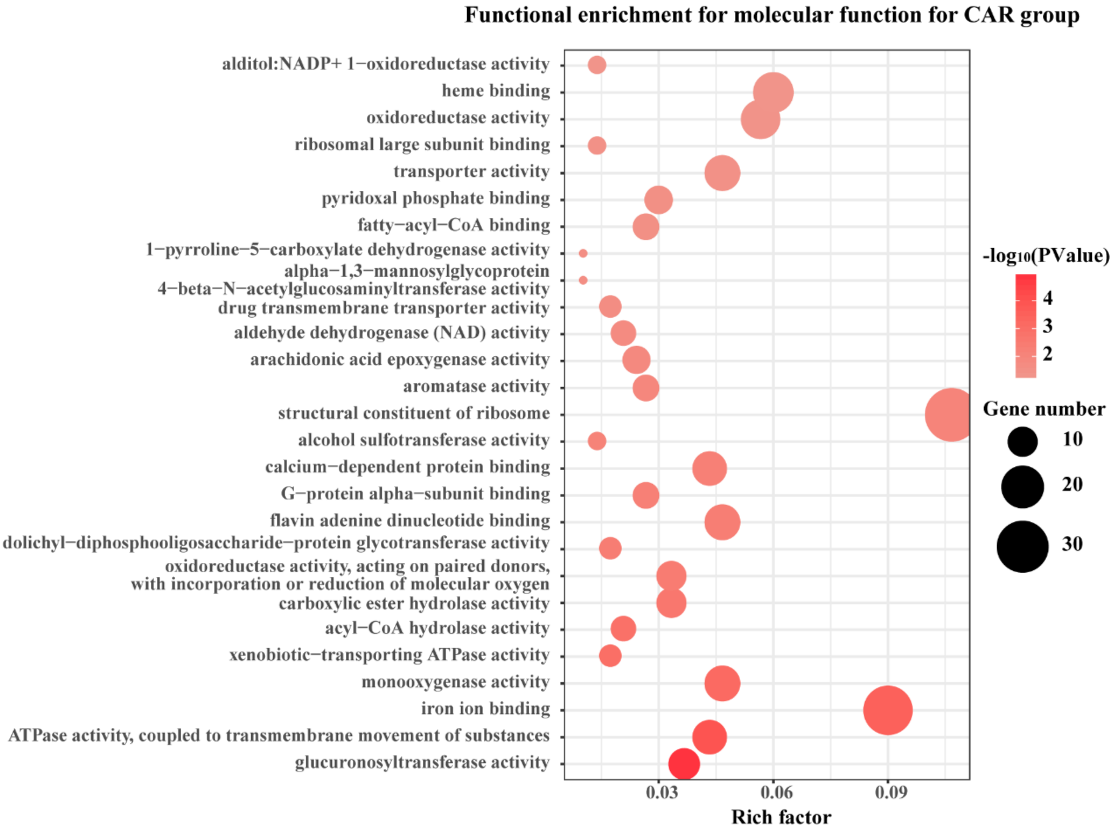

Figure S15: KEGG analysis for molecular function after CAR chemical treatment using DAVID server.

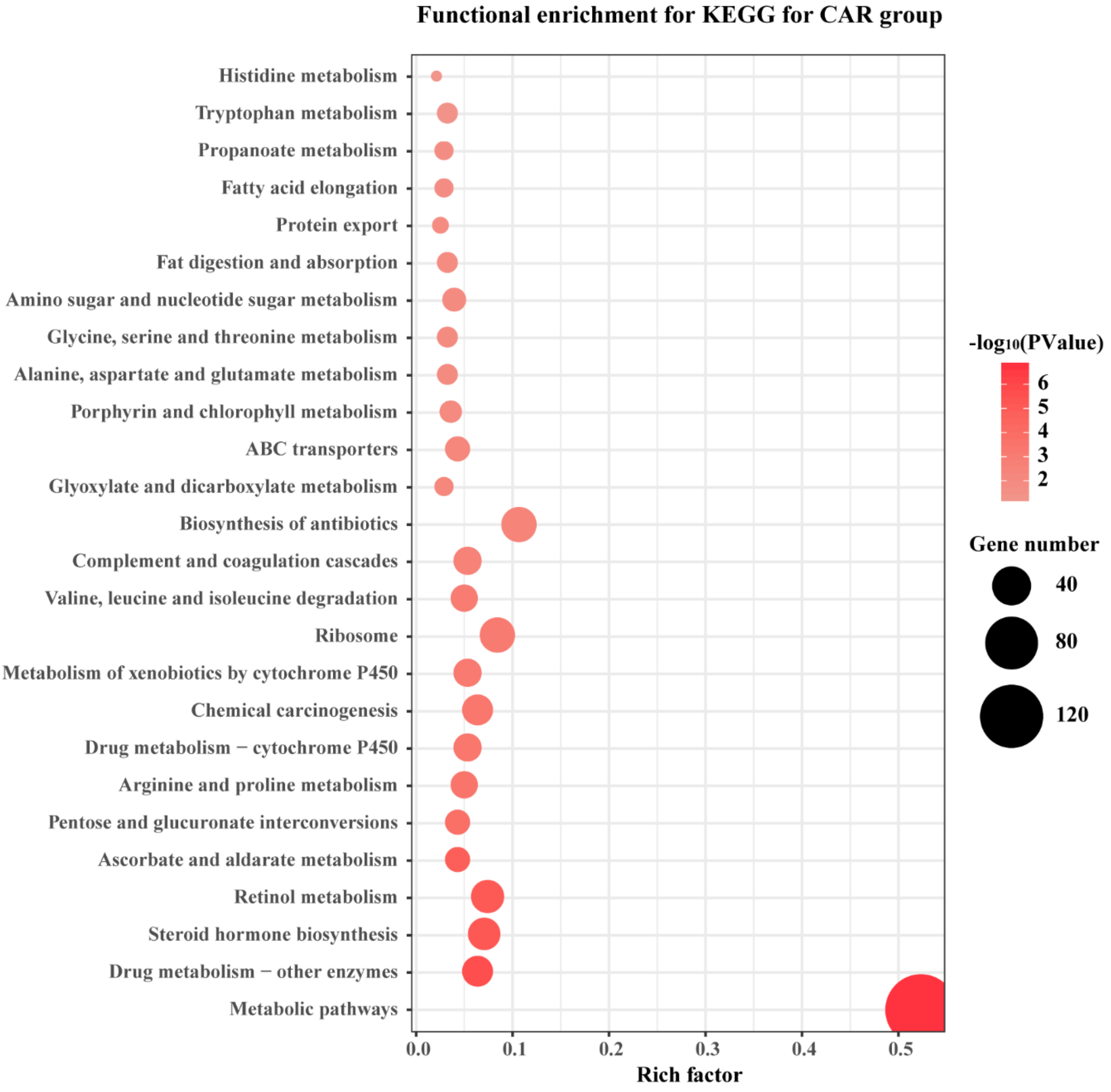

Figure S16: Functional enrichment analysis for biological process after CHL chemical treatment using DAVID server.

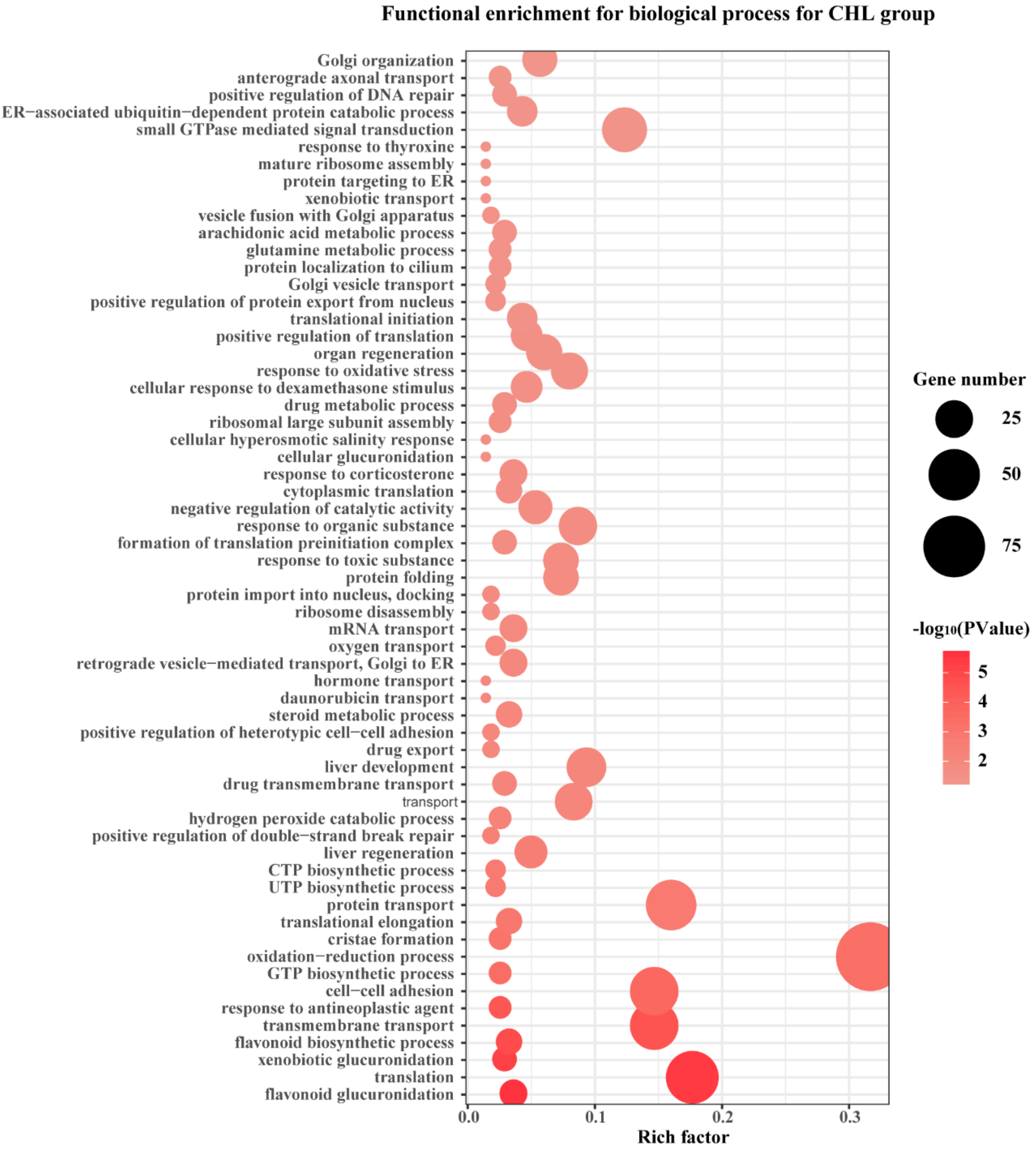

Figure S17: Functional enrichment analysis for cellular component after CHL chemical treatment using DAVID server.

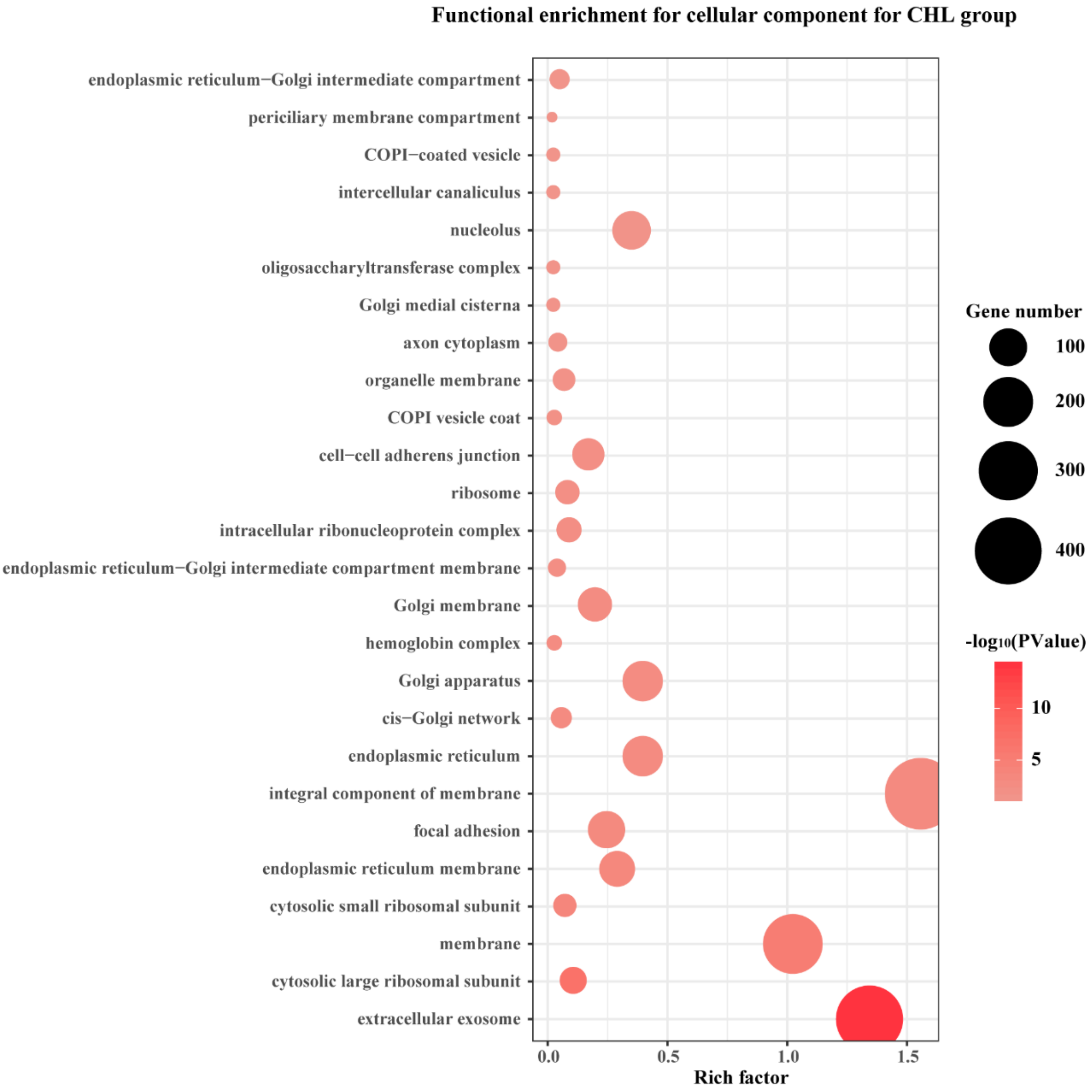

Figure S18: Functional enrichment analysis for molecular function after CHL chemical treatment using DAVID server.

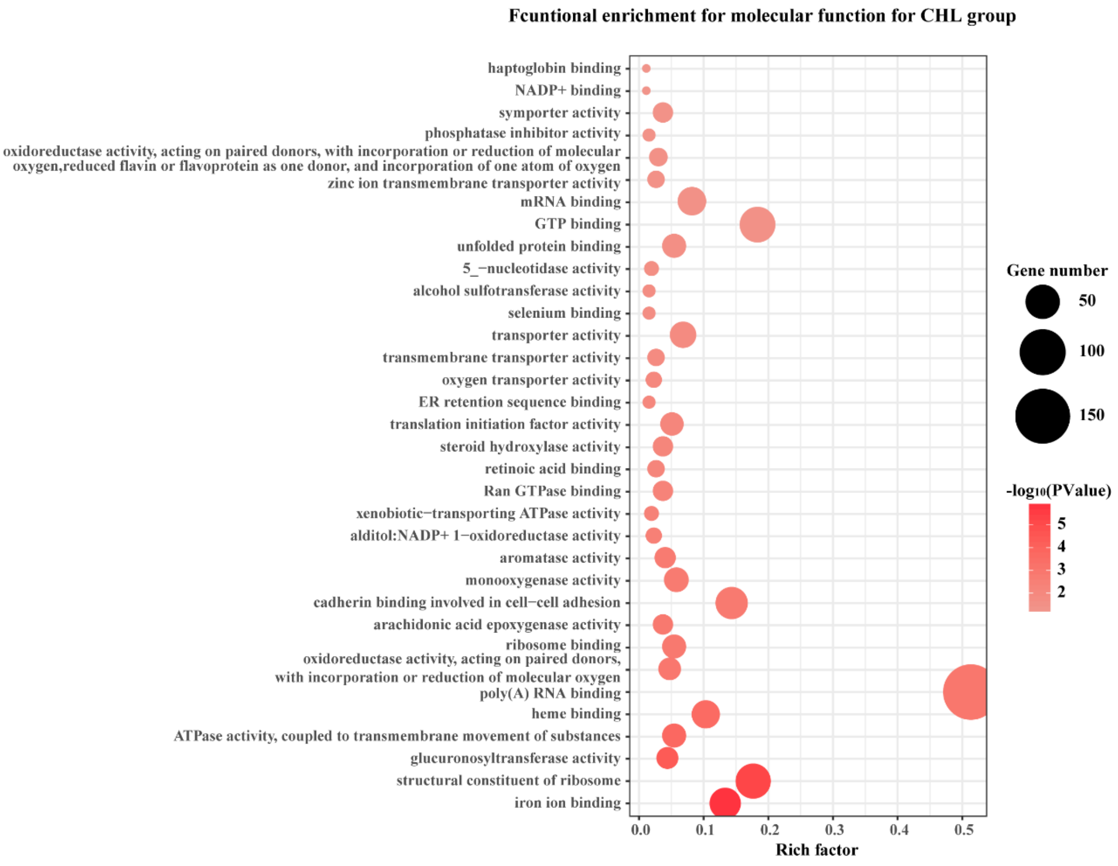

Figure S19: KEGG analysis for molecular function after CHL chemical treatment using DAVID server.

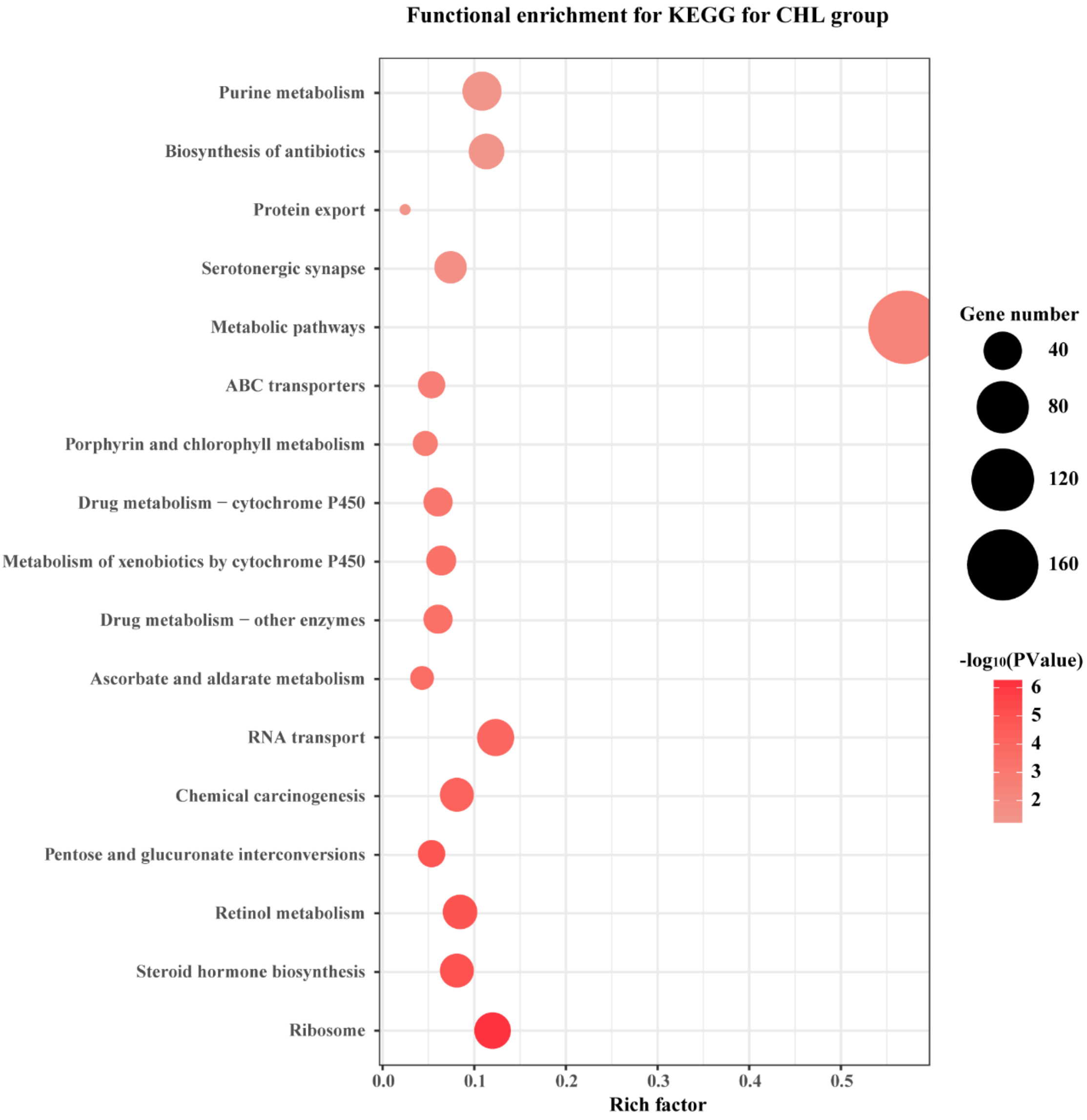

Figure S20: Functional enrichment analysis for biological process after THI chemical treatment using DAVID server.

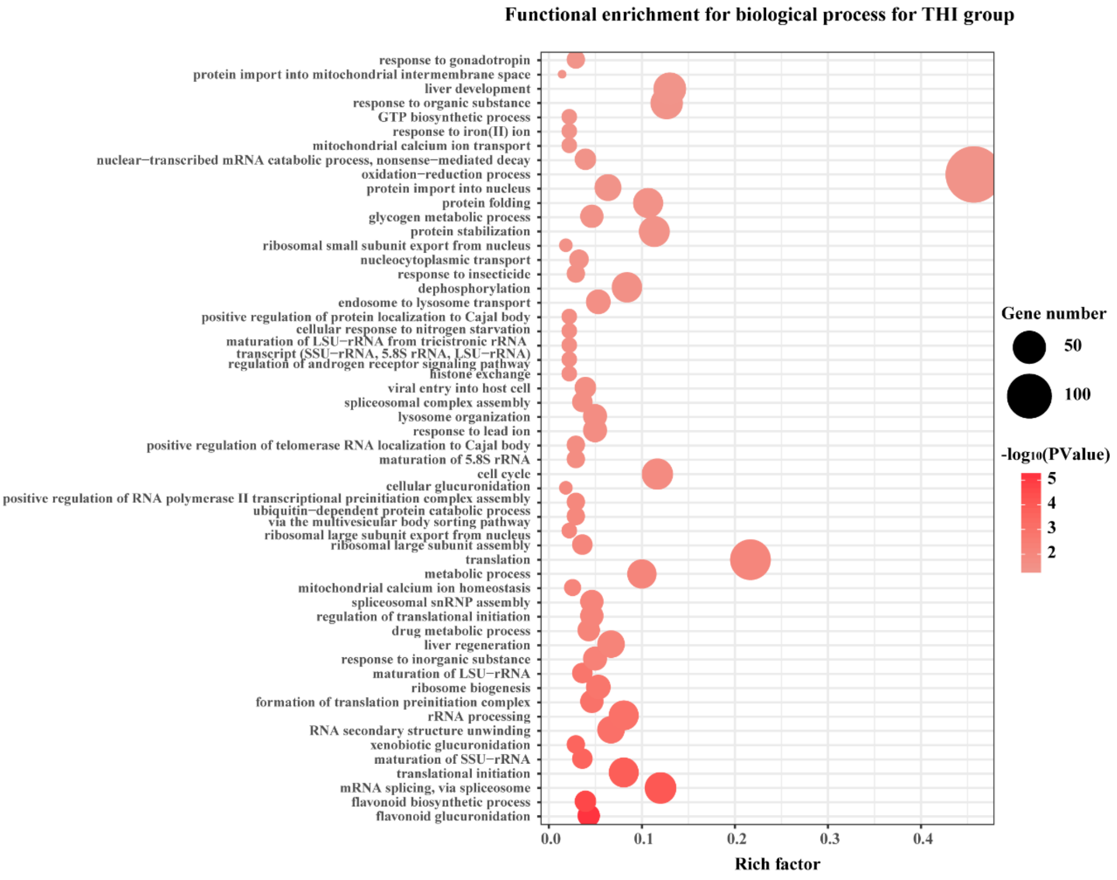

Figure S21: Functional enrichment analysis for cellular component after THI chemical treatment using DAVID server.

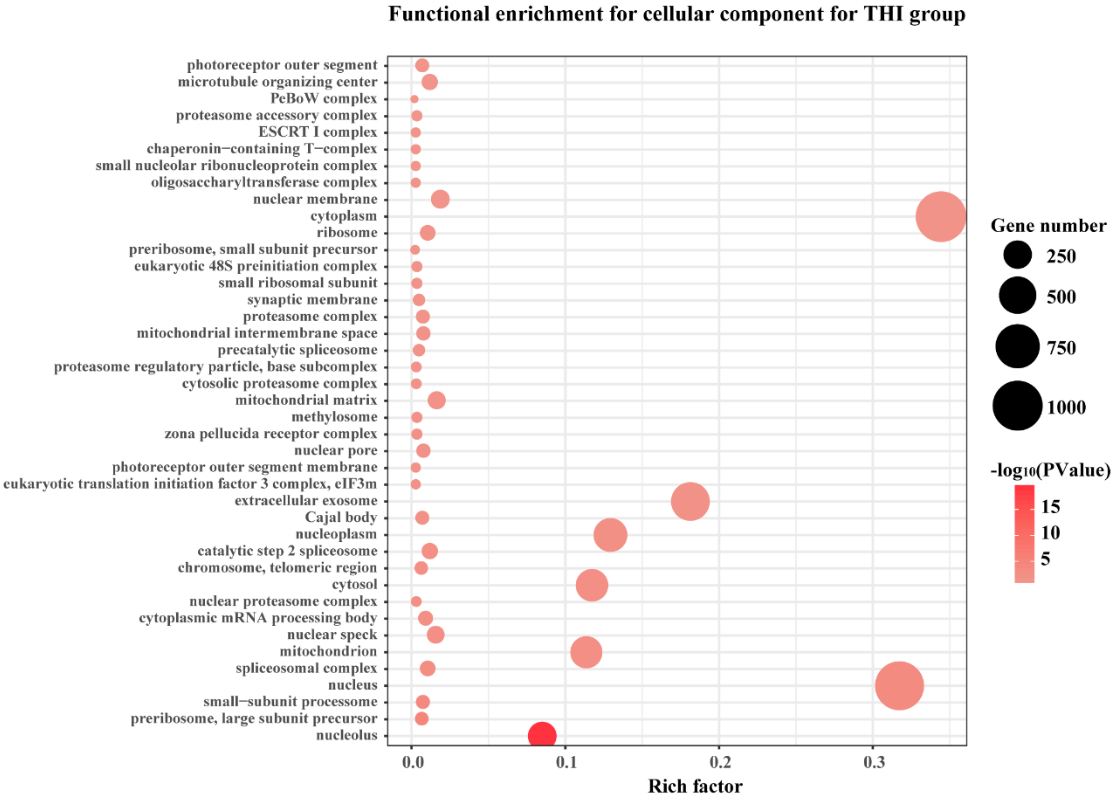

Figure S22: Functional enrichment analysis for molecular function after THI chemical treatment using DAVID server.

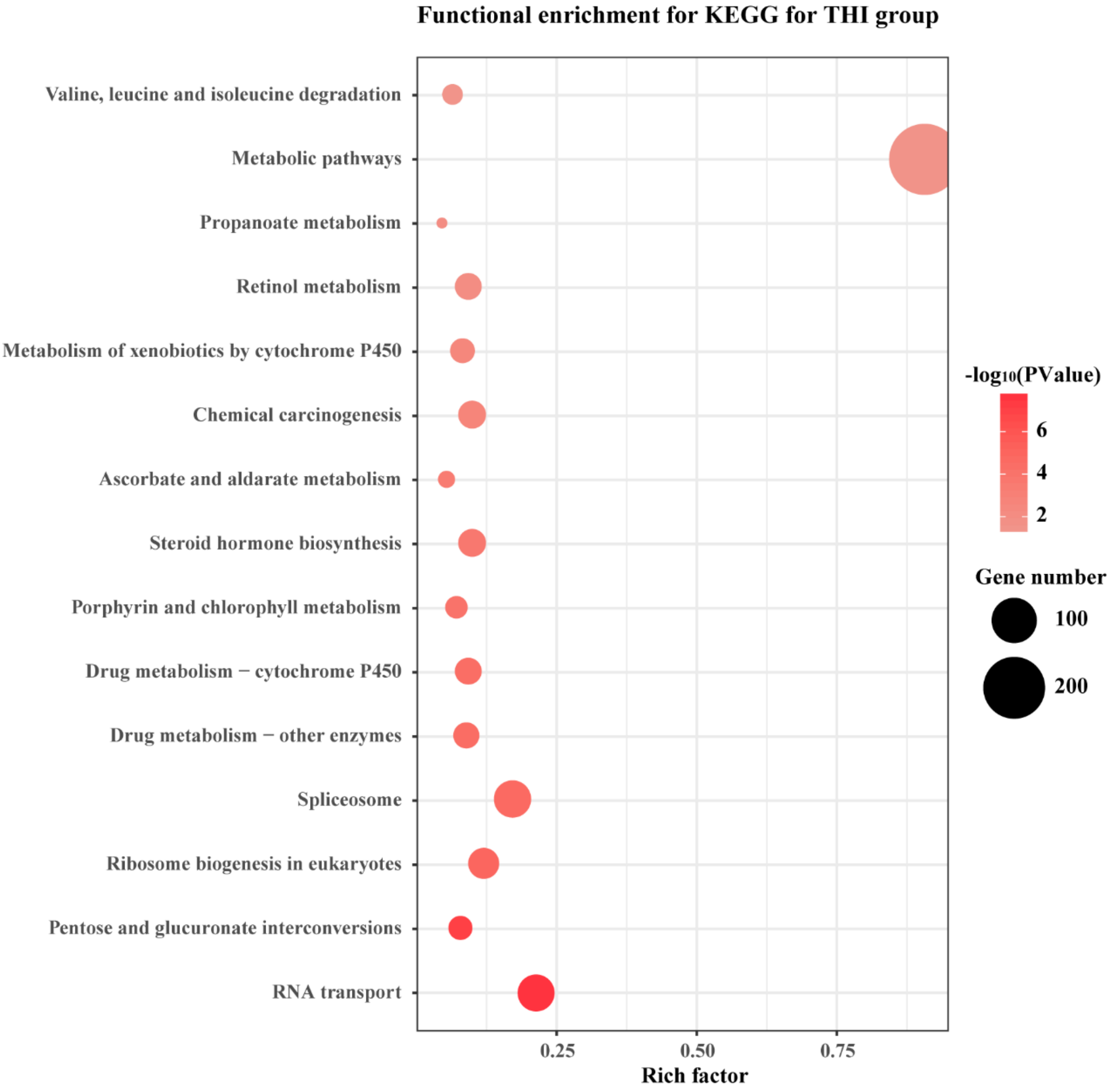

Figure S23: KEGG analysis for molecular function after THI chemical treatment using DAVID server.

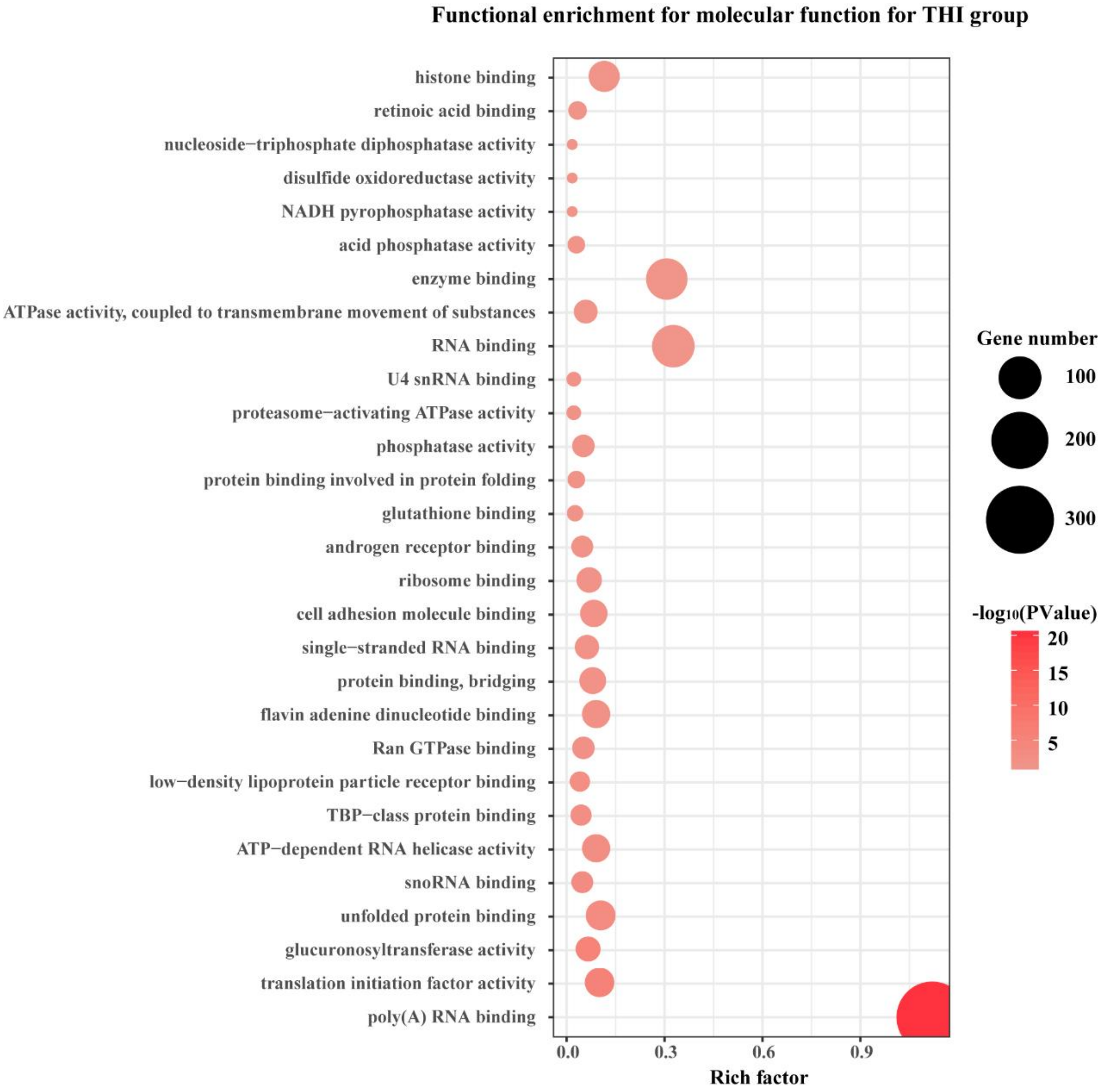

Supplement: Supplementary file 1 — Supplementary figures and table S1-3. [file ijbsv15p2296s1.pdf]
